# Supplementary material for: Multi-scale dissection, compaction and derivatization of mammalian developmental enhancers
Source: bioRxiv. 2026 Apr 21:2026.04.20.719625. Preprint. [Version 1] doi: 10.64898/2026.04.20.719625 (PMC13131614; doi:10.64898/2026.04.20.719625)
Supplement: Supplement 5 [file NIHPP2026.04.20.719625v1-supplement-5.pdf]

bioRxiv preprint doi: https://doi.org/10.64898/2026.04.20.719625; this version posted April 21, 2026. The copyright holder for this preprint (which was not certified by peer review) is the author/funder, who has granted bioRxiv a license to display the preprint in perpetuity. It is made available under aCC-BY 4.0 International license.

SUPPLEMENTARY TABLES

Table S1: List of starting developmental CREs, final model 300 bp tiles for deep dissection, and synthesized sequences (sub-tiling version 1, sub-tiling version 2, saturation mutagenesis, compaction, derivatization), together with control sequences (minP, EEF1A1p, and housekeeping controls).

Table S2: List of used oligonucleotides, plasmids, and sequencing amplicons.

Table S3: List of mapped main parietal CRM TFBS in profiled CREs (threshold normalized affinity of 0.05).

Table S4: Selected TFBS anchors for CRE derivatization (**Fig. S17**).

bioRxiv preprint doi: https://doi.org/10.64898/2026.04.20.719625; this version posted April 21, 2026. The copyright holder for this preprint (which was not certified by peer review) is the author/funder, who has granted bioRxiv a license to display the preprint in perpetuity. It is made available under aCC-BY 4.0 International license.

SUPPLEMENTARY FIGURES 1-25

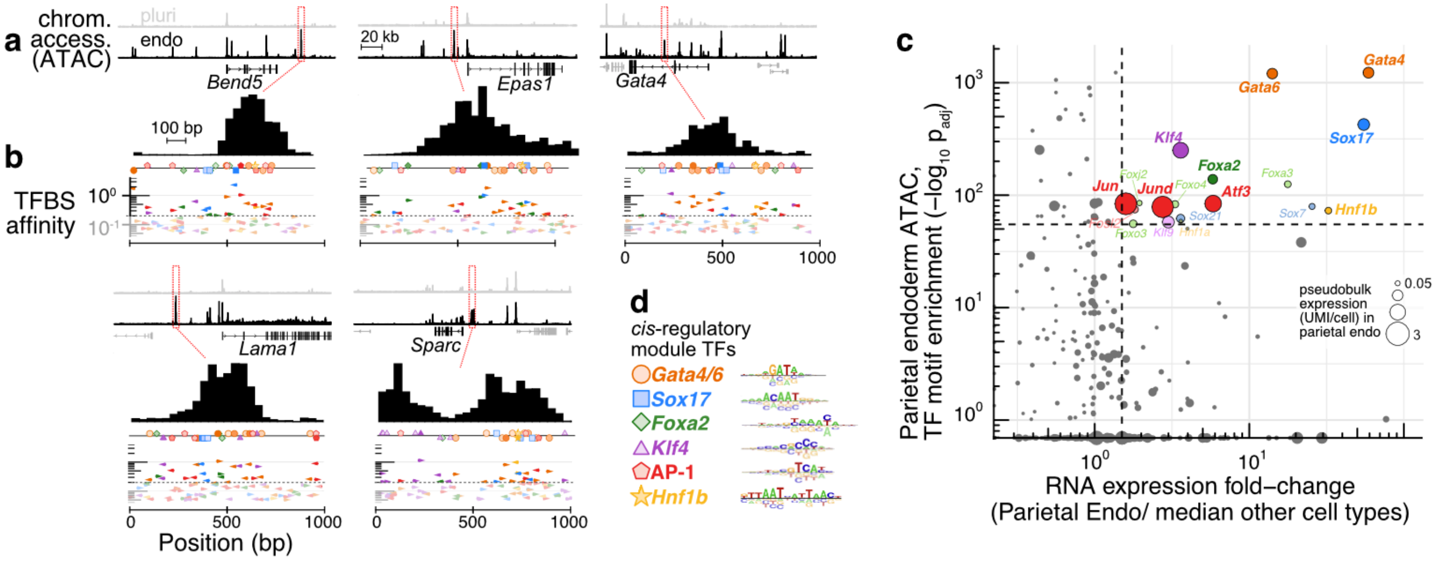

Supplementary Figure 1. Profiled parietal endoderm CREs and *cis*-regulatory module TFs. (a) Upper: pseudobulk scATAC-seq profiles[40] of pluripotent (grey) and parietal endoderm (black) cell types in mouse EBs in the vicinity of selected differentially expressed genes (200 kb windows). Profiled CREs are boxed in red. Lower: zoomed in view of the accessibility in parietal endoderm region over the full-length CRE included in the original screen[40] (≈1 kb, variable from CRE to CRE as these were originally PCR-cloned). **(b)** Arrowheads: Putative TFBSs for *cis*-regulatory module TFs, affinity mapped using quantitative binding models[74]. The x-axis (position) is aligned to the x-axis of the lower part of panel **a**. Affinities are normalized to the 99.99% score among 1M random test k-mers to uniformize scores for binding sites of different lengths. A threshold of 0.2 (dashed line) was used to select binding sites shown at the top and also in **Fig. 1b**. **(c)** Identification of the parietal endoderm *cis*-regulatory module in mouse EBs. y-axis: TFBS enrichment in parietal endoderm (-$\log_{10}$ adjusted p-value from hypergeometric enrichment in marker peaks as computed using ArchR[109] peakAnnoEnrichment function). x-axis: fold-change in pseudo-bulked mRNA expression levels of associated TF. scATAC and scRNA-seq data from mouse eEBs[40]. Highlighted TFs meet cutoffs of $-\log_{10}p_{adj} > 55$ and expression fold-change >1.5. Top members of TF paralogous families with similar binding preferences are shown with the dark hue. Pseudobulk expression level in parietal endoderm maps to point size. Most TFs have no motif enrichment ($-\log_{10}p_{adj} < 0.6$) and thus projected on or near the x-axis. **(d)** Shown are the model parameters (akin to a position weight matrix) from the MotifCentral database[74] for the CRM TFs highlighted in panel **c** (MotifCentral IDs: FOXA2: 17084, GATA4: 16735, GATA6: 17095, HNF1B: 12948, KLF4: 17150, SOX17: 10629, JUN:ATF3 [AP-1]: 15946).

bioRxiv preprint doi: https://doi.org/10.64898/2026.04.20.719625; this version posted April 21, 2026. The copyright holder for this preprint (which was not certified by peer review) is the author/funder, who has granted bioRxiv a license to display the preprint in perpetuity. It is made available under aCC-BY 4.0 International license.

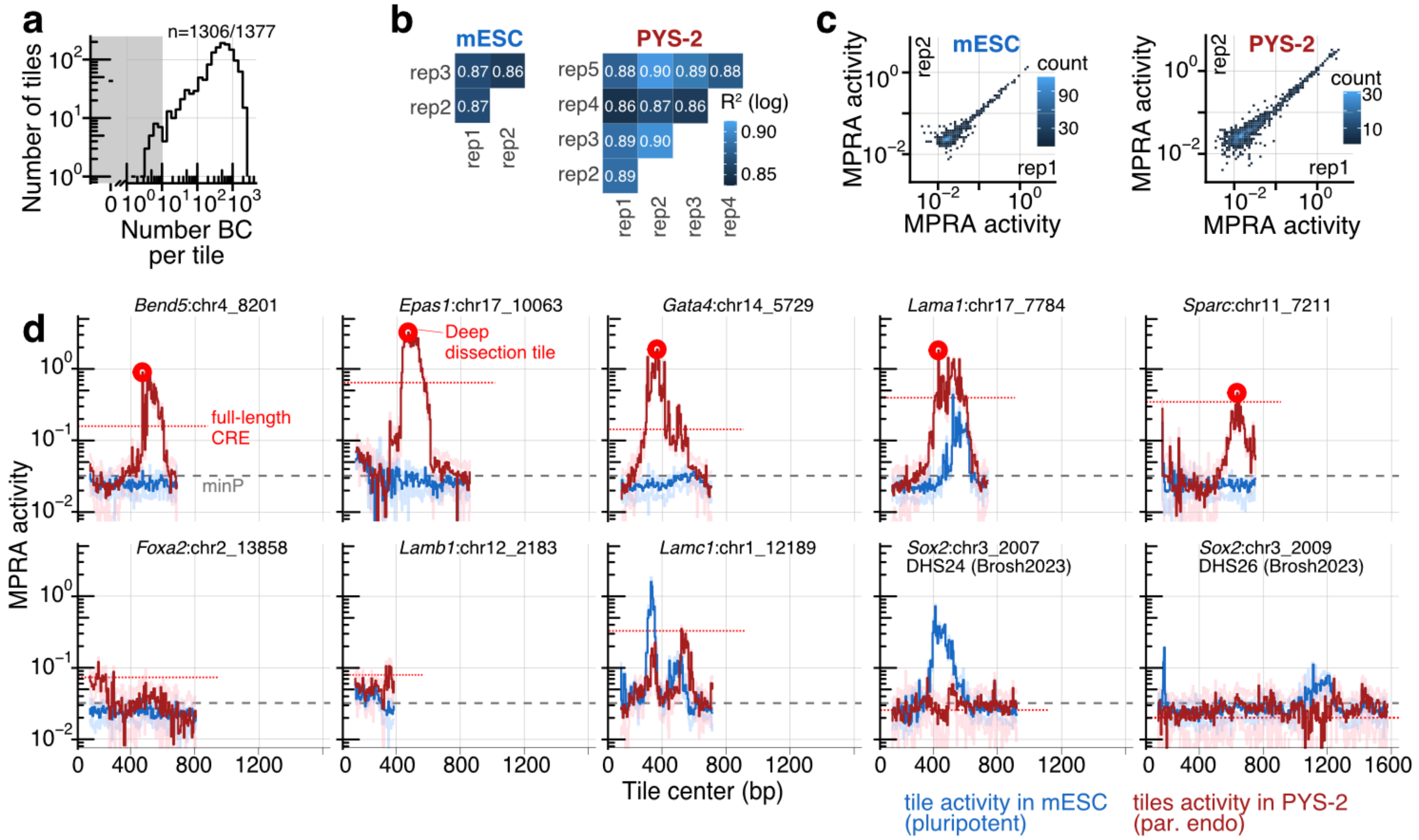

Supplementary Figure. 2. Single-size (270 bp), single-orientation sub-tiling of 10 developmental CREs in two cell lines. (a) Number of BCs per tile in the MPRA library dictionary of 270 bp tiles scanning across 10 developmental CREs identified by a scQer performed in mouse EBs[40]. Total library complexity: 658.6k BCs for 1377 tiles, with a median of 492 BCs/tile and n=1306/1377 with ≥10 associated BCs. **(b-c)** Reproducibility for episomal MPRA data in mESCs and PYS-2 cells across biological replicates, including Pearson's R^2 on log-transformed activities (panel **b**) and representative scatter plots (panel **c**). **(d)** MPRA data from the sub-tile scan (270 bp tiles) with a stride of 5 bp for the 10 developmental CREs, which are of different sizes. These include, from left-to-right then top-to-bottom, 7 specific to parietal endoderm, 1 bi-functional, and 2 specific to pluripotent cells, from the mouse EB scQer study[40]. Median activity across replicates per sub-tile position is shown (red: PYS-2, blue: mESC). Pale lines delineate standard deviation and min/max across replicates in PYS-2 and mESC respectively. The full-length versions of the 5 enhancers on the top row exhibited strong endoderm-specific activity in our scQer screen in mouse EBs[40], while the full-length versions of the *Sox2*-control-region enhancers (*Sox2*:chr3_2007 and *Sox2*:chr_2009) were inactive in PYS-2 cells. Tiles used for fine dissection (saturation mutagenesis, compaction, derivatization) were taken as the 300 bp tiles centered on the maximum PYS-2 activity 270 bp tiles from each of the 5 enhancers on the top row (light red points). Notably, these all had activity >15-fold above the basal minP-only control in PYS-2, and were below the minP control in mESC cells.

bioRxiv preprint doi: https://doi.org/10.64898/2026.04.20.719625; this version posted April 21, 2026. The copyright holder for this preprint (which was not certified by peer review) is the author/funder, who has granted bioRxiv a license to display the preprint in perpetuity. It is made available under aCC-BY 4.0 International license.

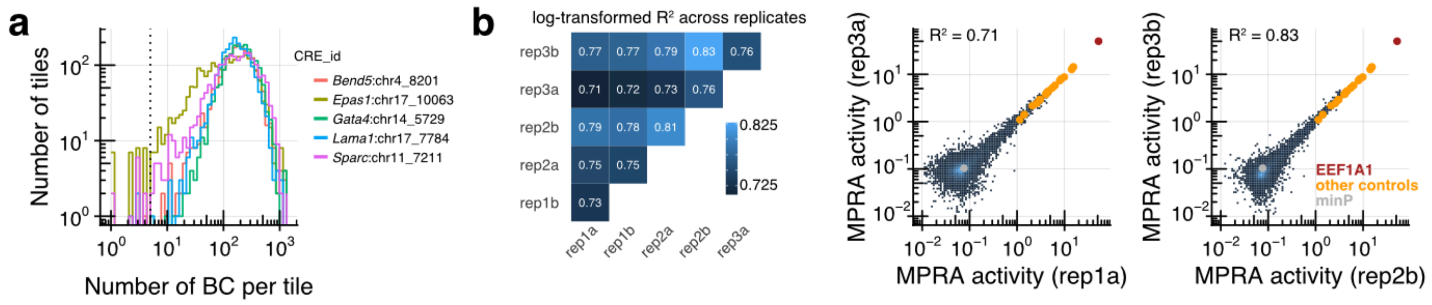

Supplementary Figure 3. Quality metrics for the multi-sized sub-tiling MPRA library. (a) Distribution of BC counts per sub-tile (defined as a regulatory sub-fragment specified by tile size, genomic position, and orientation), stratified by CRE. The final reporter library recovered the vast majority of all possible sub-tiles (9,959 of 10,108; 98.4%) with ≥5 BCs per sub-tile, comprising 1.91 million total BCs. The interquartile range of BC counts per sub-tile was 94-253. **(b)** Reproducibility of MPRA activity per sub-tile across biological replicates. The heatmap reports pairwise R^2 values computed on log-transformed activity for all replicate pairs. The replicate pairs with the lowest and highest correlations are shown at right. Control promoters are highlighted for reference (minP-only in grey, the IGVF-20-promoter control set in orange, and the *EEF1A1* promoter in red).

bioRxiv preprint doi: https://doi.org/10.64898/2026.04.20.719625; this version posted April 21, 2026. The copyright holder for this preprint (which was not certified by peer review) is the author/funder, who has granted bioRxiv a license to display the preprint in perpetuity. It is made available under aCC-BY 4.0 International license.

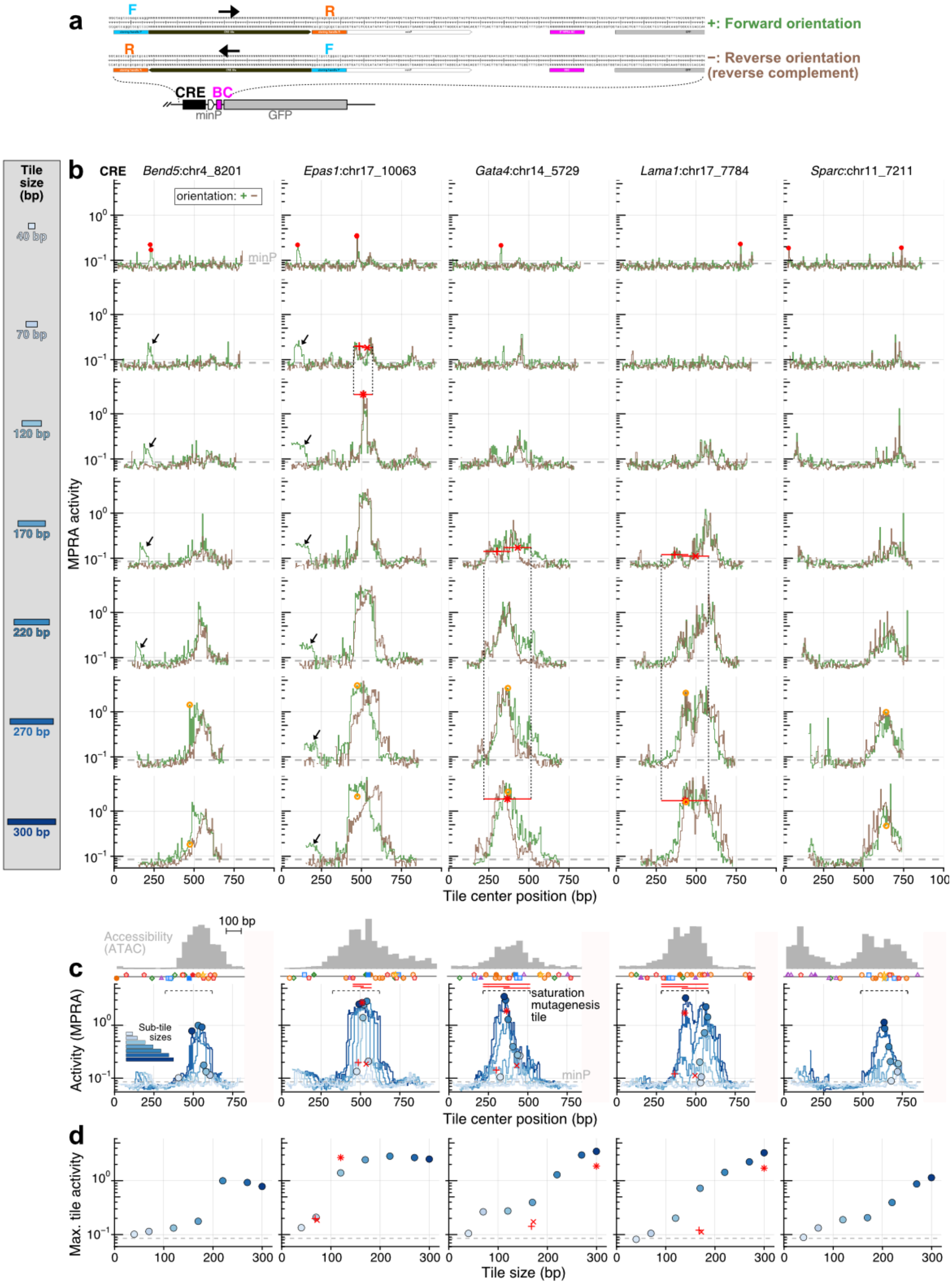

Supplementary Figure 4. Dense, multi-sized, both-orientation sub-tiling MPRAs of high-activity developmental CREs. (a) Schematic of 5′ MPRA construct that illustrates how CRE sub-tiles were cloned in both forward and reverse orientations. Each CRE sub-tile contained distinct left and right 15-bp cloning handles (F and R) used for amplification from the oligo pools. Reverse-orientation constructs were generated using primers with swapped homology arms. Thus,

bioRxiv preprint doi: https://doi.org/10.64898/2026.04.20.719625; this version posted April 21, 2026. The copyright holder for this preprint (which was not certified by peer review) is the author/funder, who has granted bioRxiv a license to display the preprint in perpetuity. It is made available under aCC-BY 4.0 International license.

tiles in each orientation are flanked by different handles relative to the minimal promoter (minP). Consequently, we cannot fully exclude the possibility that chimeric junctions between CRE sequence and cloning handles create additional TF binding sites that contribute to measured activity, although we consider this unlikely. **(b)** Unfiltered data from dense, multi-sized sub-tiling MPRAs of all five CREs, shown separately for forward (green) and reverse (brown) orientations. Each column corresponds to a CRE and each row to a tile size. The grey dashed line indicates the mean activity of the minP-only control. Red points in the 40-bp row denote the 9 tiles with activity >2-fold above minP background. Black arrows indicate extended regions of activity outside major chromatin accessibility peaks; of note, these were overwhelmingly orientation-specific. Red symbols (+, ×, ✳) mark the short and long synergizing tiles highlighted in **Fig. 2**. Orange points indicate the maximum-activity 270-bp tiles from our initial MPRA screen (**Fig. S2**) that were selected for saturation mutagenesis after extension by 15 nt on each side to generate a 300-bp tile; the activity of the resulting 300-bp tiles is shown in the bottom row. In one instance (*Bend5*:chr4_8201), the extended tile had drastically lower activity (see also **Fig. S9**). **(c-d)** Similar to **Fig. 2b-c**, but for all five CREs, and ordered horizontally in line with panel **b** above.

bioRxiv preprint doi: https://doi.org/10.64898/2026.04.20.719625; this version posted April 21, 2026. The copyright holder for this preprint (which was not certified by peer review) is the author/funder, who has granted bioRxiv a license to display the preprint in perpetuity. It is made available under aCC-BY 4.0 International license.

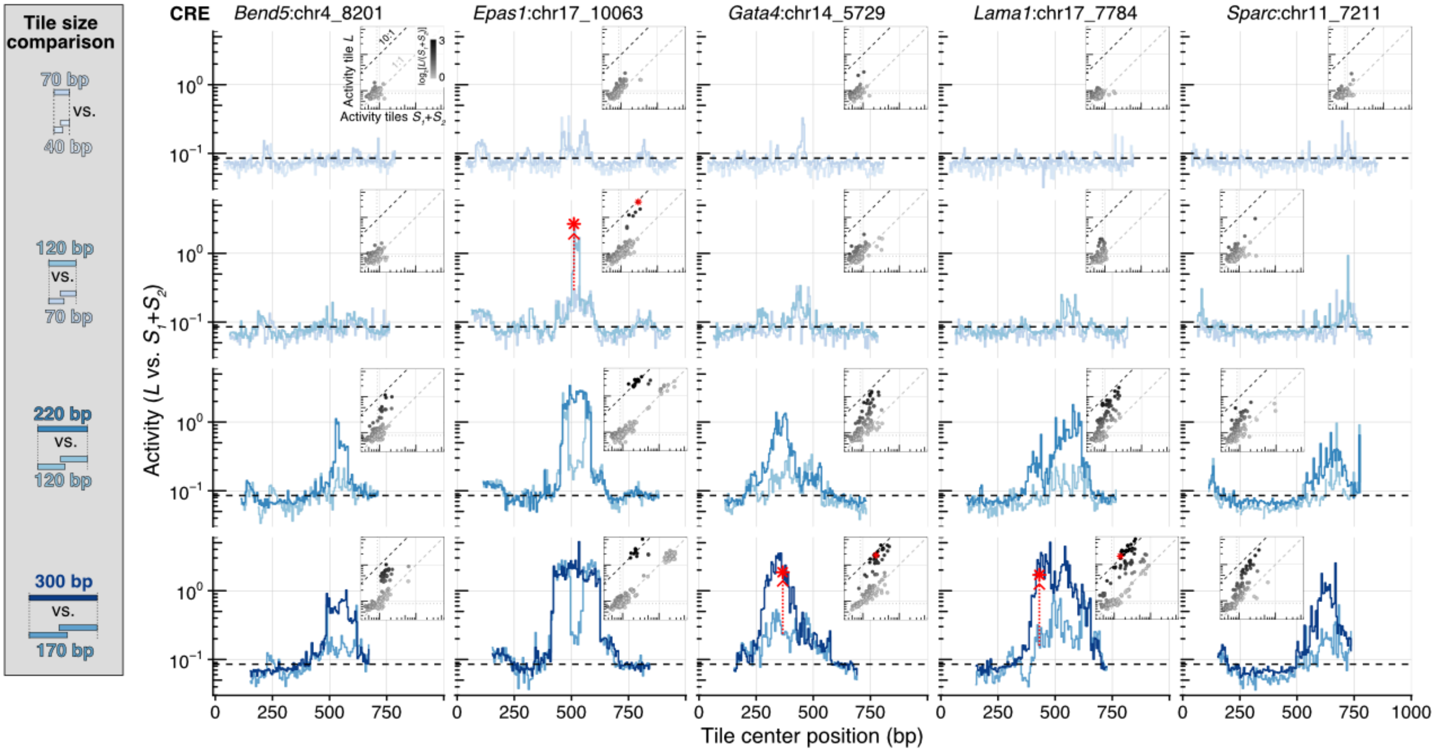

Supplementary Figure 5. Sliding-window comparisons of matched short and long tiles reveal synergistic CRE sub-tiles. Similar to **Fig. 2d**, but shown for all CREs (columns) and for multiple short/long tile size pairings (rows: 40+40 vs. 70 bp, 70+70 vs. 120 bp, 120+120 vs. 220 bp, 170+170 vs. 300 bp), as illustrated to the left. For each comparison, two adjacent short tiles (S_1 and S_2) are matched to the corresponding long tile (L) spanning the same genomic interval. Red points mark the tiles highlighted with red symbols in **Fig. 2b-c**, and red arrows below these points that indicate the fold synergy of the long tile (L) relative to the summed activity of the two short tiles (S_1+S_2). Each inset shows a direct comparison of S_1+S_2 vs. L activity, where points deviating from the diagonal indicate synergistic sub-tiles. Color scale used for points in insets is shown only in the top left panel. Grey and black dashed lines in insets correspond to 1:1 (no synergy) and 10:1 (strong synergy).

bioRxiv preprint doi: https://doi.org/10.64898/2026.04.20.719625; this version posted April 21, 2026. The copyright holder for this preprint (which was not certified by peer review) is the author/funder, who has granted bioRxiv a license to display the preprint in perpetuity. It is made available under aCC-BY 4.0 International license.

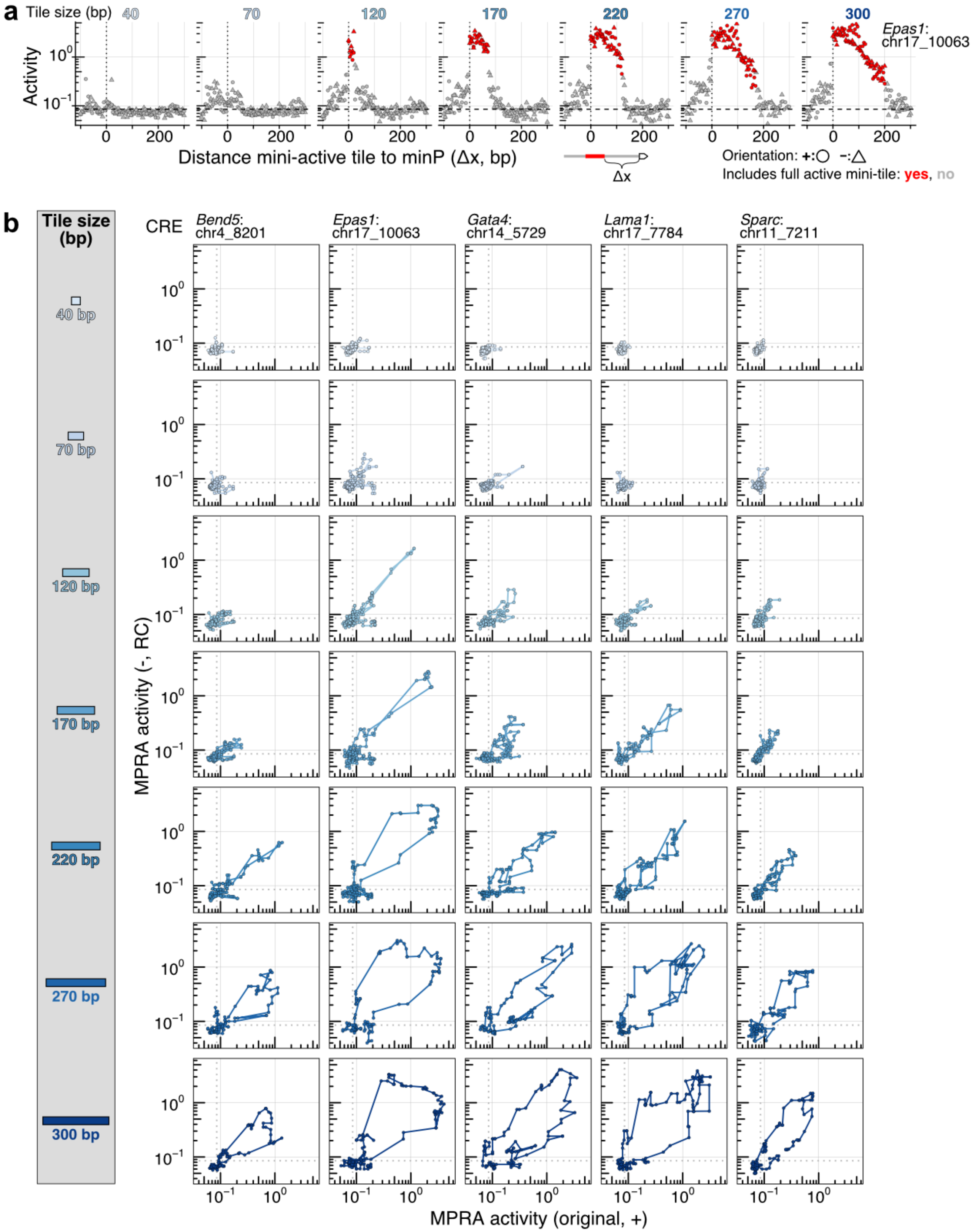

Supplementary Figure 6. Multi-sized sub-tiling reveals orientation-dependent short-range positional effects in MPRAs. (a) Re-plot of the sub-tiling MPRA data for CRE *Epas1*:chr17_10063, with activity shown as a function of the distance between minP and the active mini-tile, rather than absolute tile position. Forward (circles) and reverse (triangles) orientation tiles collapse onto a common distance–activity curve, indicating that distance to the promoter explains a

bioRxiv preprint doi: https://doi.org/10.64898/2026.04.20.719625; this version posted April 21, 2026. The copyright holder for this preprint (which was not certified by peer review) is the author/funder, who has granted bioRxiv a license to display the preprint in perpetuity. It is made available under aCC-BY 4.0 International license.

substantial fraction of the variability between orientations. Red points denote tiles that fully contain the active mini-tile, whereas grey points correspond to tiles that do not. **(b)** Direct comparison of MPRA activity for matched forward (+) and reverse (–) orientation tiles. Each panel corresponds to a different CRE (columns) and tile size (rows). The progressive opening of the forward/reverse trajectories at larger tile sizes indicates increasing orientation-dependent differences for longer fragments, consistent with a length-dependent positional effect on enhancer-minP activity in this reporter context.

bioRxiv preprint doi: https://doi.org/10.64898/2026.04.20.719625; this version posted April 21, 2026. The copyright holder for this preprint (which was not certified by peer review) is the author/funder, who has granted bioRxiv a license to display the preprint in perpetuity. It is made available under aCC-BY 4.0 International license.

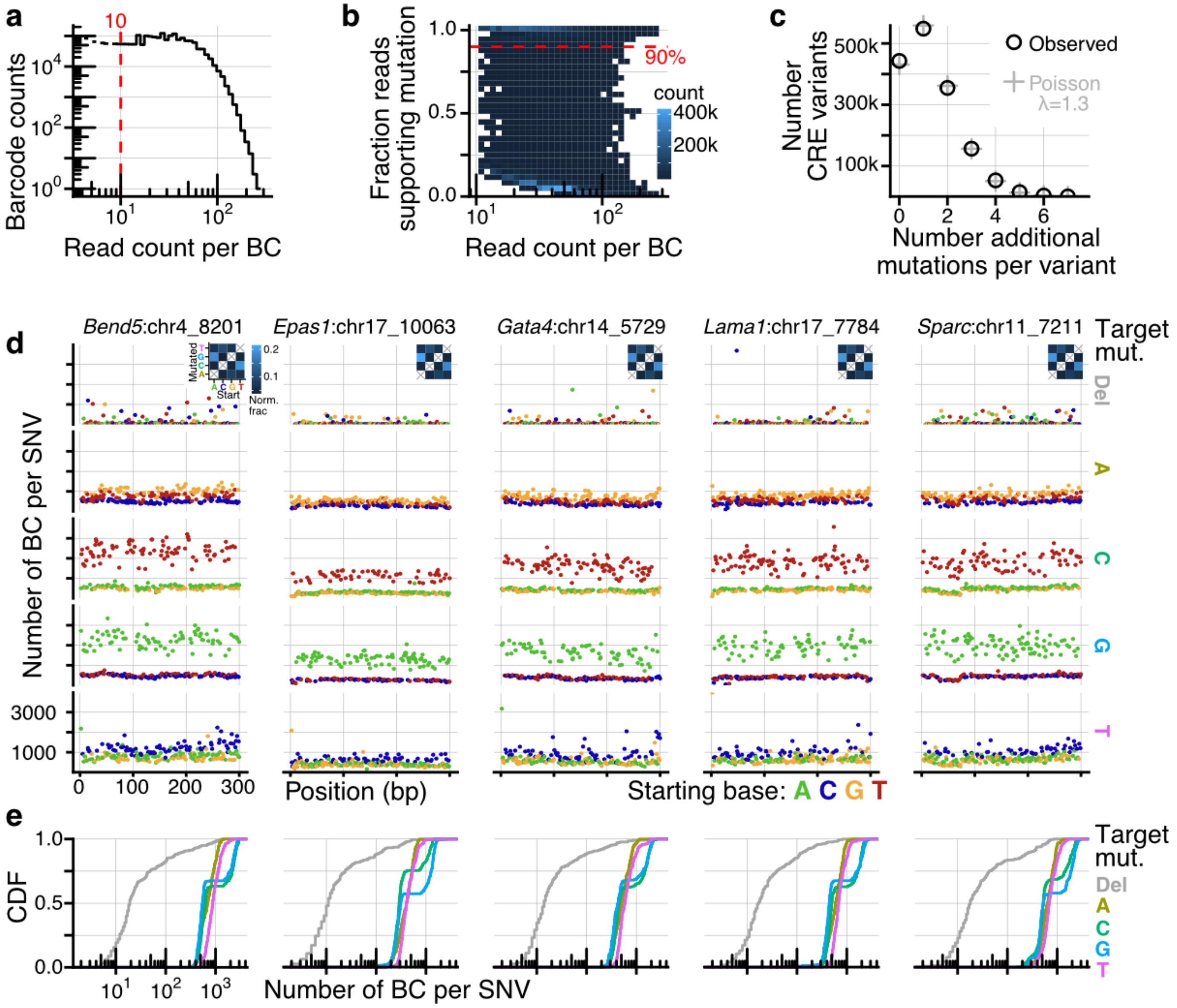

Supplementary Figure 7. Quality control and characterization of multi-hit saturation mutagenesis library.
(a) Distribution of read count per BC for the saturation mutagenesis MPRA library. The final library consisted of 1.6M barcodes (with >10 reads in the variant-to-BC association phase). **(b)** Two-dimensional histogram (color indicating density) showing read count associated with a given BC vs. fraction of reads supporting a specific mutation. Mutation-to-BC associations were called if >90% of reads supported the mutation (red dashed line). **(c)** Distribution of additional mutations relative to what was programmed (*i.e.* # mutations minus 1) mapped in the variant-to-BC dictionary. The observed distribution (black circles 'o') closely matches the Poisson distribution (grey crosses '+'), as expected for a random fixed rate mutagenesis. Mean of distribution is λ=1.3, corresponding average additional mutation rate of 0.4% per base from the PCR amplification and cloning process. **(d)** Characterization of the mutational spectrum: number of mapped barcodes with a given mutation (rows: different target mutation, columns: different CREs, colors: starting base). Taq-based polymerase preferences are immediately manifest (A:T→G:C) and add mutations over an otherwise constant baseline as expected from commercially synthesized libraries with a single programmed point mutation per sequence. Inset shows quantification of the mutational spectrum using a normalized fraction accounting for base composition. **(e)** Cumulative distribution of barcode per target mutation ID. Cs and Gs BC counts are bimodal as a result of the putative PCR-derived mutations manifesting itself in only ⅓ possible contexts.

bioRxiv preprint doi: https://doi.org/10.64898/2026.04.20.719625; this version posted April 21, 2026. The copyright holder for this preprint (which was not certified by peer review) is the author/funder, who has granted bioRxiv a license to display the preprint in perpetuity. It is made available under aCC-BY 4.0 International license.

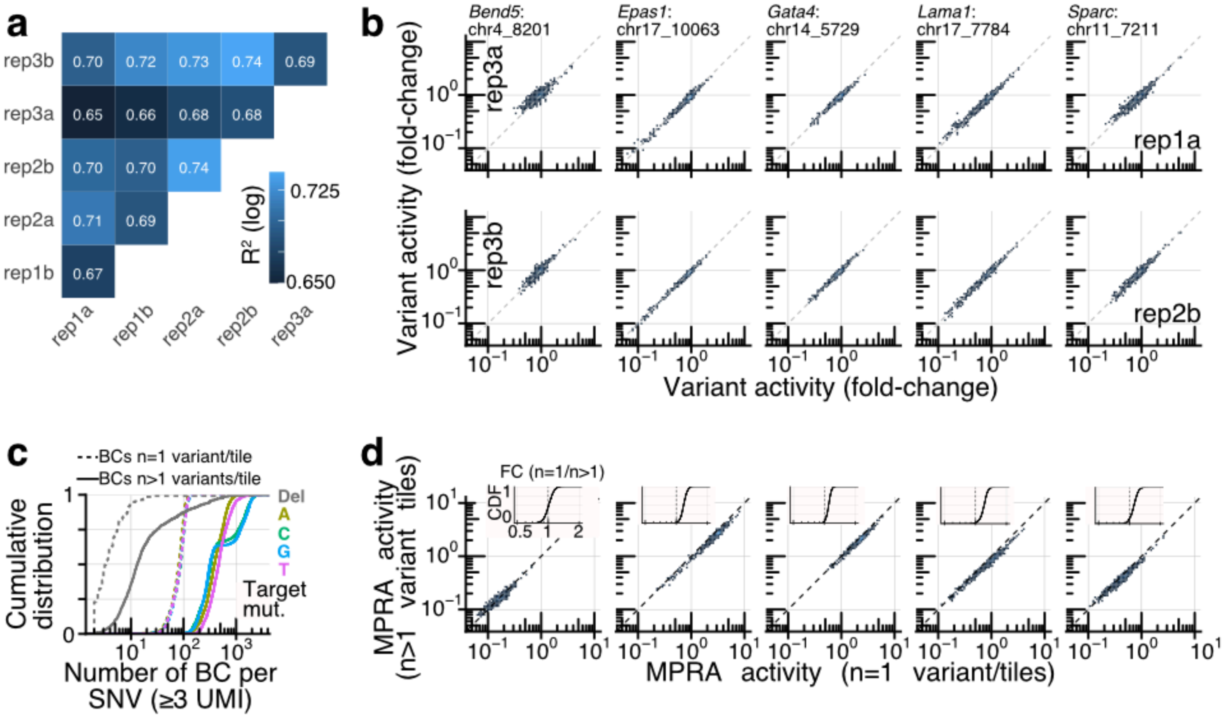

Supplementary Figure 8. Saturation mutagenesis MPRA data quality metrics. (a) Reproducibility of MPRA activity for saturation mutagenesis across replicates. The heatmap reports pairwise R^2 values computed on log-transformed variant effects for all replicate pairs, *i.e.* 2 technical replicates for each of three biological replicates. **(b)** Variant effects comparisons for replicate pairs with the lowest and highest correlations. Each column corresponds to a different CRE and each row is a different comparison (top: rep1a vs. rep3a, bottom: rep2b vs. rep3b). **(c)** Distribution of number of BC per target mutation (with at least 3 UMIs mapped in the dictionary) in the library, stratified by whether the tile has a single mutation ('n=1' tiles) vs. multiple mutations ('n>1' tiles). As a result of the inferred PCR mutagenesis, most CRE variants bear multiple mutations. **(d)** Comparison of variant effect estimated from 'n=1' (x-axes) vs. 'n>1' (y-axes) tiles. Each column corresponds to a different CRE as in panel **b**. Inset shows cumulative distribution of fold-change between 'n=1' vs. 'n>1' tiles, showing a slight systematic shift to lower values in the 'n>1' tiles. In panels **b** and **d**, deletions are not included to avoid overt counting variability given their low representation in the library (see panel **c** and **Fig. S7e**).

bioRxiv preprint doi: https://doi.org/10.64898/2026.04.20.719625; this version posted April 21, 2026. The copyright holder for this preprint (which was not certified by peer review) is the author/funder, who has granted bioRxiv a license to display the preprint in perpetuity. It is made available under aCC-BY 4.0 International license.

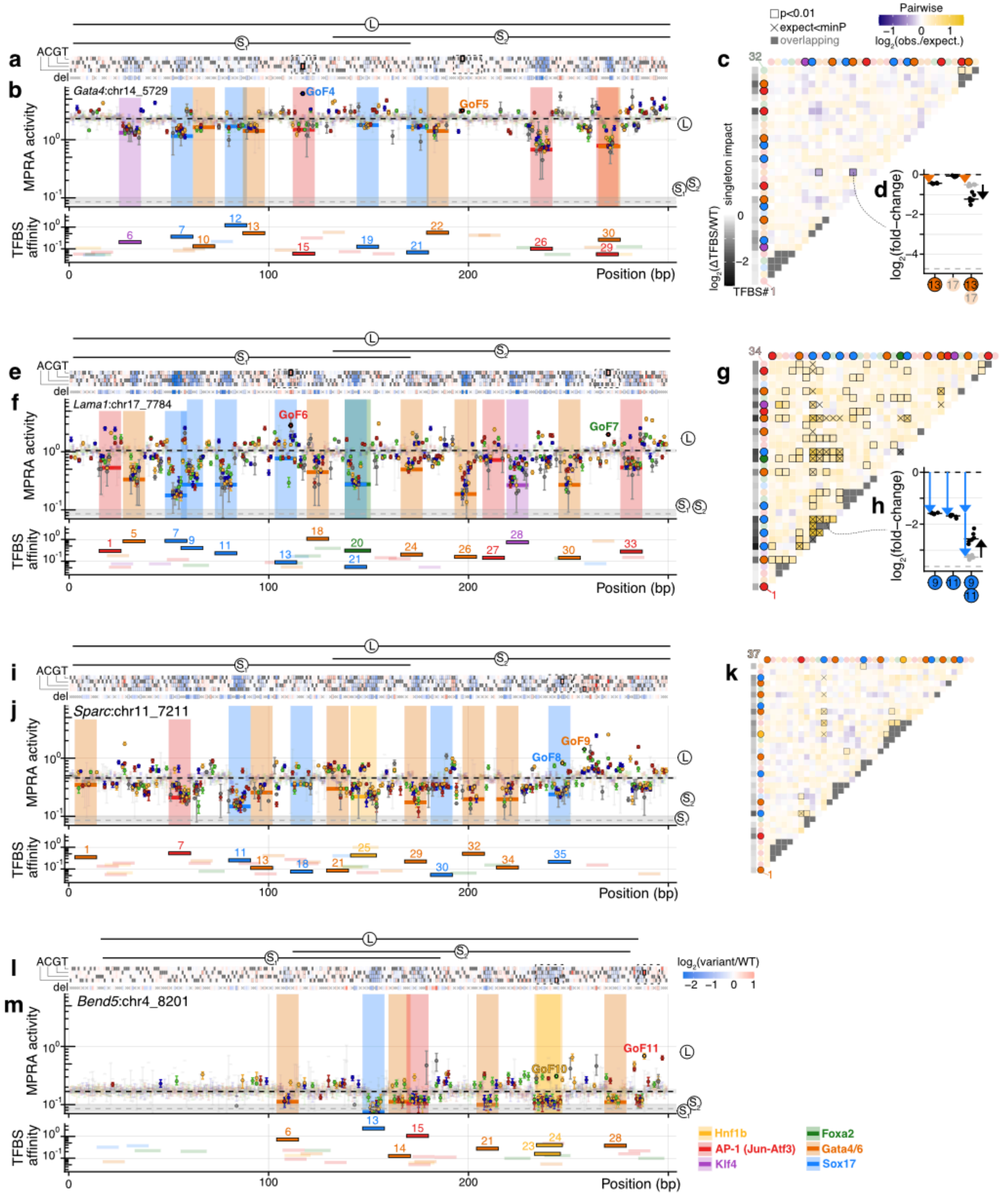

Supplementary Figure 9. High-resolution saturation mutagenesis TFBS maps for four of the model CREs. Similar to **Fig. 3**, but here for the *Gata4*:chr14_5729 (panels **a-d**), *Lama1*:chr17_7784 (panels **e-h**), *Sparc*:chr11_7211 (panels **i-k**), and *Bend5*:chr4_8201 (panels **l, m**) CREs. The activity of the mutagenized tile for the *Bend5:chr4_8201* CRE was too close to the minP baseline to allow mapping of epistatic interactions.

bioRxiv preprint doi: https://doi.org/10.64898/2026.04.20.719625; this version posted April 21, 2026. The copyright holder for this preprint (which was not certified by peer review) is the author/funder, who has granted bioRxiv a license to display the preprint in perpetuity. It is made available under aCC-BY 4.0 International license.

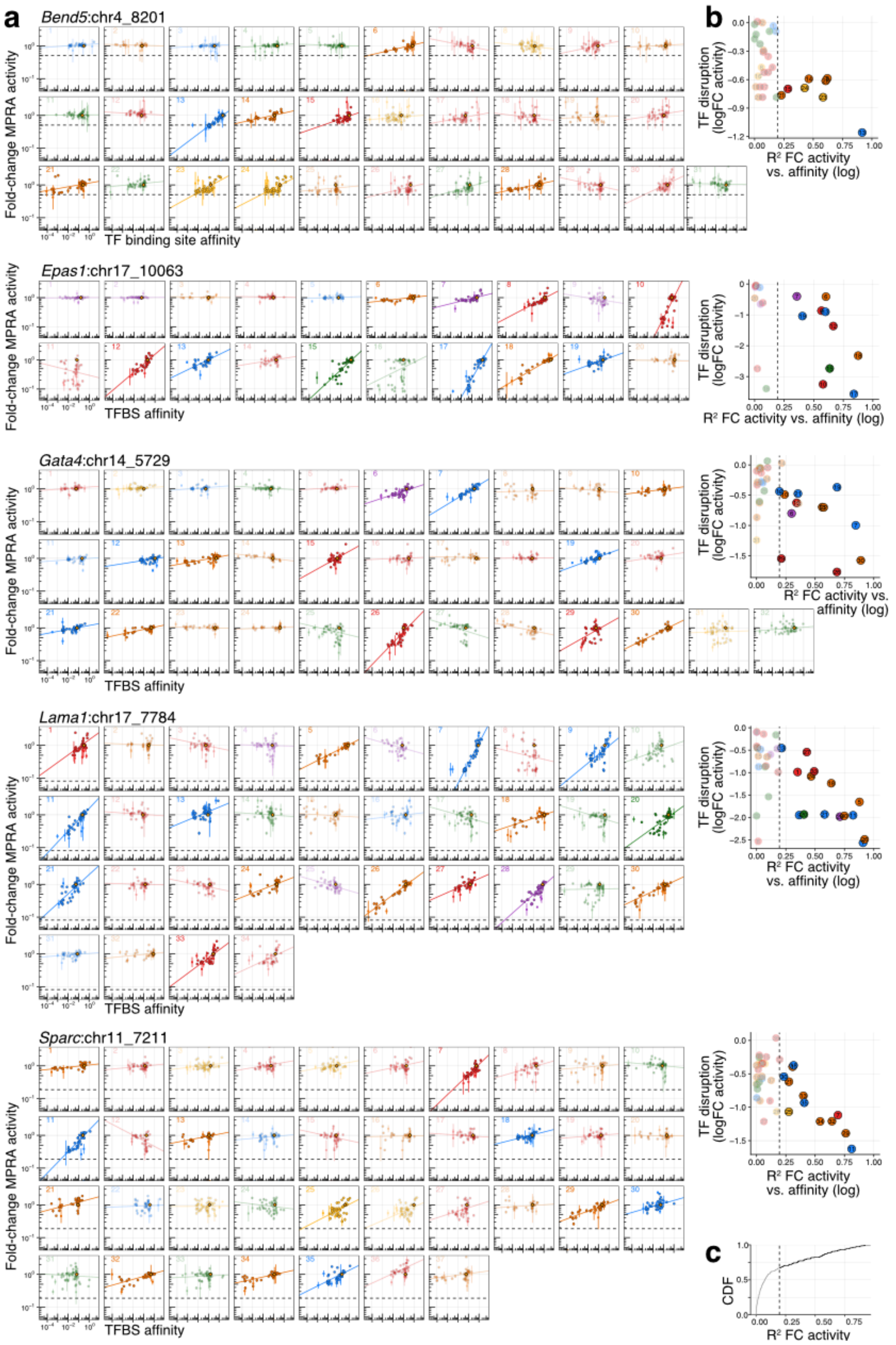

Supplementary Figure 10. Affinity vs. activity correlation for putative binding sites of parietal endoderm TFs. **(a)** Multi-panel view of correlation between variant effect predicted affinity (x-axes) vs. measured activity (y-axes) for mutations within mapped TFBS, organized by CREs. Each plot corresponds to mapped TFBS (numbering follows positional order from left to right along the sequence). Each point in each panel corresponds to a mutation of the TFBS. WT sequences are marked with yellow diamonds. Errorbar spans the standard deviation across replicates. Line corresponds to linear regression on the log-transformed variables. Non-singleton-functional TFBS are shaded in a paler hue. Colors for different TFs match those of **Fig. 1b**. The expected fold-change to minP only is shown as a horizontal dashed line. **(b)** Plot per CRE showing the relationship between the fold-change in activity from disrupting the TFBS (y-axes) vs. the R^2 correlation in the log-transformed plots shown in panel **a** (x-axes). Binding site color and numbering follow those of panel **a**. Singleton-functional TFBS are defined as those with R^2>0.195 (dashed line). **(c)** Cumulative distribution of R^2 across all TF binding sites illustrating the threshold used to designate singleton-functional TFBS.

bioRxiv preprint doi: https://doi.org/10.64898/2026.04.20.719625; this version posted April 21, 2026. The copyright holder for this preprint (which was not certified by peer review) is the author/funder, who has granted bioRxiv a license to display the preprint in perpetuity. It is made available under aCC-BY 4.0 International license.

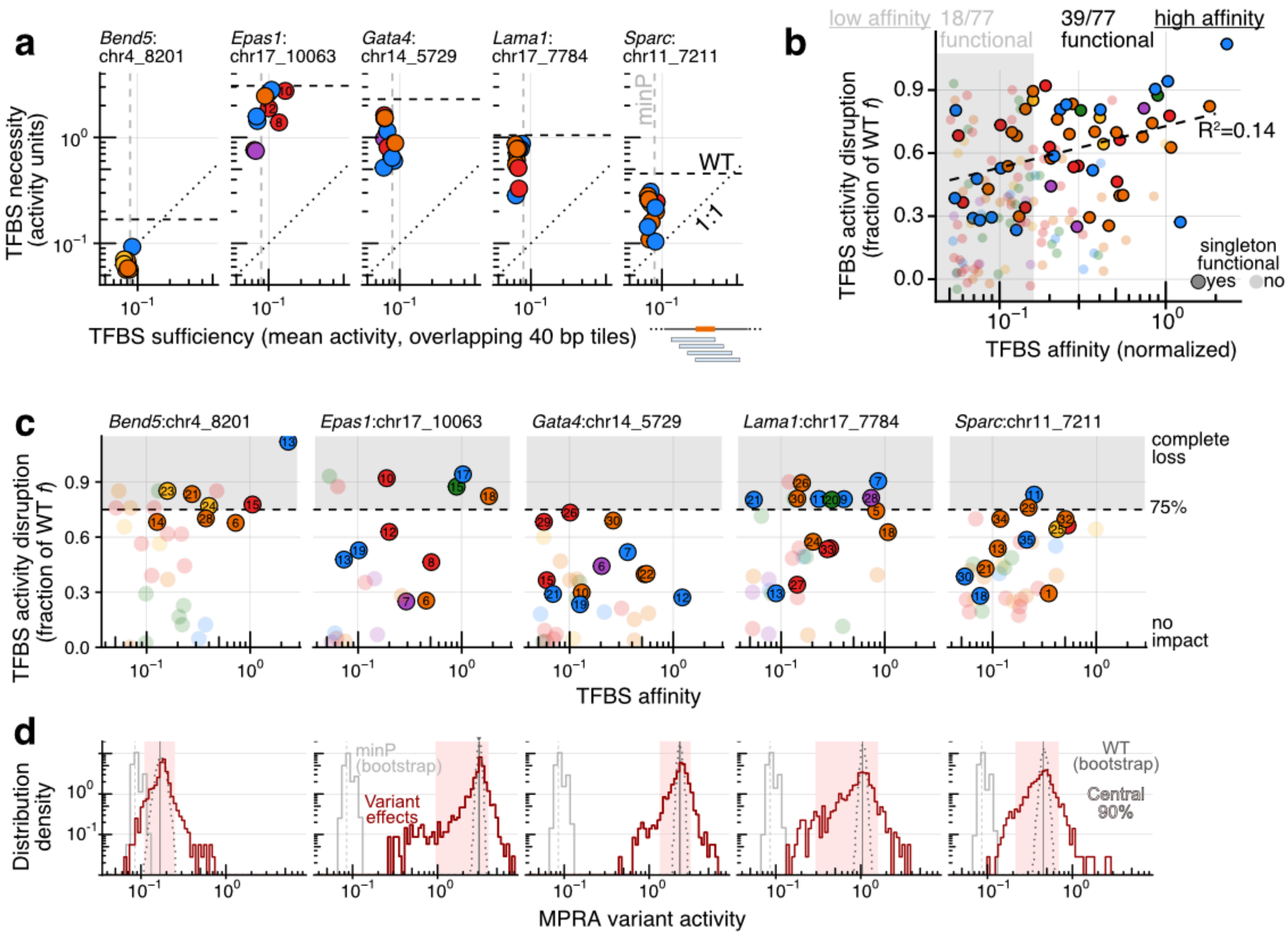

Supplementary Figure 11. Sufficiency & necessity of TFBSs for activity and variant effect distribution. (a) Quantification of the sufficiency (x-axes: log-scaled average activity of all 40 bp tiles bearing the TFBS from sub-tiling experiment shown in **Fig. 2**) vs. necessity (y-axes: log-scaled activity disruption from panel **b**) of individual TFBS within each of the model CREs. **(b)** Necessity scores of TFBSs (*y*-axis) are only weakly predicted by affinity (*x*-axis). Necessity scores are quantified as in **Fig. 3h**, but plotted here for all TFBSs mapped to parietal endoderm CRM TFs. Singleton-functional sites are marked with a dark edge. Although affinity correlates only weakly with functional importance, higher-affinity sites are ~2-fold enriched for singleton-functional TFBSs (Fisher's exact test: $p < 0.0007$). **(c)** Quantification of necessity of individual TFBS (y-axes: log-scaled activity disruption: fraction of WT *f* lost upon disruption) as a function of affinity (x-axis). Grey zone delineates binding sites that abrogate >75% of WT CRE activity upon disruption. Binding site numbering and shading follows **Figs. 3b**, **S9b,f**,**j**,**m**. **(d)** Distribution of MPRA activity variant effects (dark red) for each of the model CREs (ordered as in panel **b**). WT activity is marked with a vertical black line. Bootstrap resampling distribution of the WT sequences matching the coverage of mutations within the respective CRE is shown in dotted grey line. Central 90% of variant effects is shown as a light pink shading. Bootstrap resampling for minP is shown as a solid gray line (same subsampling for all sub-panels).

bioRxiv preprint doi: https://doi.org/10.64898/2026.04.20.719625; this version posted April 21, 2026. The copyright holder for this preprint (which was not certified by peer review) is the author/funder, who has granted bioRxiv a license to display the preprint in perpetuity. It is made available under aCC-BY 4.0 International license.

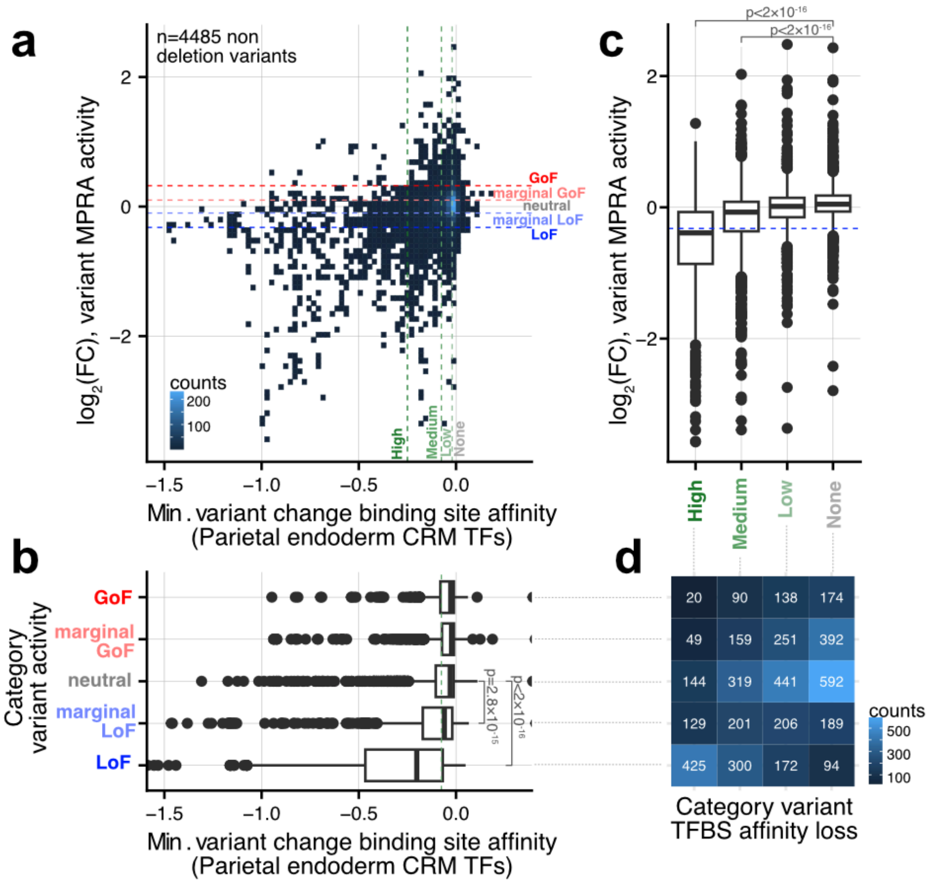

Supplementary Figure 12. Change in TFBS affinity vs. change in TFBS activity for loss-of-function mutations. (a) Two-dimensional histogram of correlation between the maximal decrease in normalized affinity $\delta_{\min}$ to parietal endoderm CRM TFs (x-axis; Gata4/6, Sox17, Klf4, Foxa2, AP-1, Hnf1b) vs. variant effect (y-axis; $\log_2$ fold-change activity) for every possible substitution in all five model CREs. Categorizations used in panels **b-d** are indicated by dashed lines. To calculate $\delta_{\min}$, systematic mapping of TFBS is done and the CRM TF associated with the largest decrease in normalized affinity at that position is assigned. **(b)** Boxplot showing $\delta_{\min}$ values stratified into categorized functional impact of the mutation (neutral: $|\log_2 FC|<0.1$, marginal LoF: $-0.32<\log_2 FC<-0.1$, LoF: $\log_2 FC < -0.32$, marginal GoF: $0.1<\log_2 FC<0.32$, GoF: $\log_2 FC> 0.32$). Marginal LoF and LoF categories are associated to significantly more TFBS disruptions as quantified by the maximal decrease in normalized affinity $\delta_{\min}$ (one-sided Wilcoxon test $p<10^{-14}$ compared to neutral mutations). Concretely, ~75% of LoF variants (>1.25-fold decrease) are associated with $\delta_{\min}< -0.075$ (vertical dashed line). **(c)** Converse plot of functional impact stratified by nature of TFBS disruption (none: $\delta_{\min}>-0.02$, low: $-0.075< \delta_{\min}<-0.02$, medium: $-0.25< \delta_{\min}<-0.075$, high: $\delta_{\min}< -0.25$). Medium/high disruptions to affinity lead, on average, to significantly lower activity than non-disruptive mutations (one-sided Wilcoxon test: $p < 10^{-15}$). **(d)** Heatmap showing the number of mutations in the different bi-dimensional categorizations, *i.e.* the relationship between the extent of loss-of-affinity (columns; four categories aligned to panel **c** above) and the change-of-function (rows; four categories aligned to panel **b** to left).

bioRxiv preprint doi: https://doi.org/10.64898/2026.04.20.719625; this version posted April 21, 2026. The copyright holder for this preprint (which was not certified by peer review) is the author/funder, who has granted bioRxiv a license to display the preprint in perpetuity. It is made available under aCC-BY 4.0 International license.

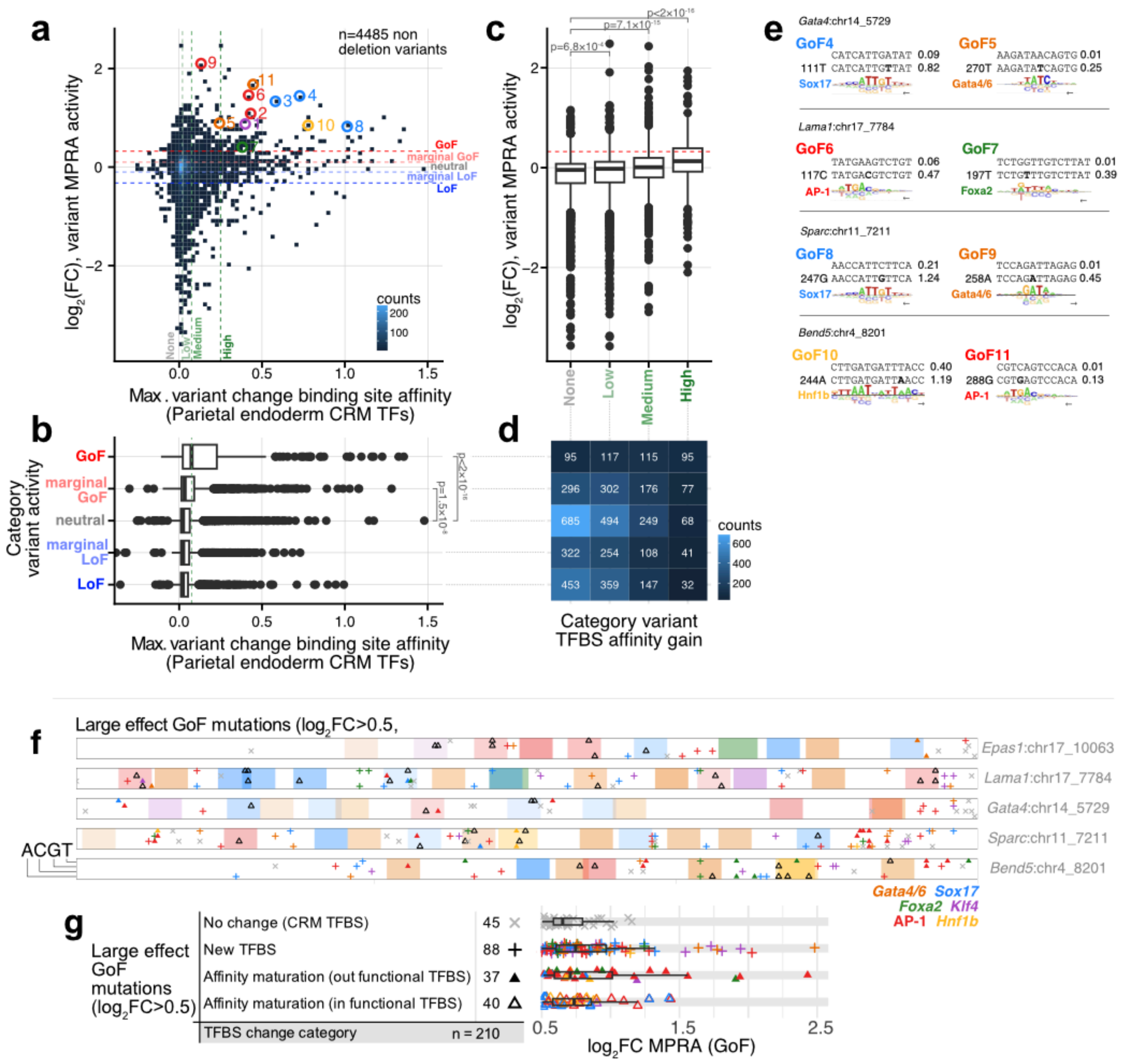

Supplementary Figure 13. Change in TFBS affinity vs. change in TFBS activity for gain-of-function mutations. (a-d) Similar to **Fig. S12**, but here focused on the maximal increase in normalized affinity δ_{max} to parietal endoderm CRM TFs. In panel **a**, specific GoF mutations highlighted in **Fig. 3** and **S9** are marked. **(e)** Same as **Fig. 3d**, but here for the 8 GoF mutations highlighted in **Fig. S9**. One example per CRE was selected to be related to affinity-maturation (left column) and another to *de novo* TFBS creation (right column, corresponding to mutation in the core segment of the motif). **(f)** Heatmap summarizing the distribution of large GoF mutations ($log_2FC > 0.5$; n = 210) for all five CREs (rows). The locations of singleton-functional TFBSs are delineated by shading, with hues map to TFBS identity (key lower right) and intensities to necessity scores. The locations of GoF mutations are marked by different symbols based on the nature of the TFBS changes: grey x: change not mappable to a CRM TFBS; open coloured triangles: affinity-maturation of a singleton-functional TFBS (class i in text); full coloured triangle: affinity-maturation of a non-singleton-functional TFBS (class ii in text); colored +: creation of new TFBS (class iii in text). Colors of symbols correspond to the associated TF, and their vertical position to the identity of the substitution. **(g)** Summary of the number of GoF mutations in each mechanistic category shown in panel **f**. Effect-size distributions for corresponding rows of the GoF categories shown at right. No pairwise comparison between categories was significant (two-sided Wilcoxon rank sum test with Bonferroni correction: p > 0.42).

bioRxiv preprint doi: https://doi.org/10.64898/2026.04.20.719625; this version posted April 21, 2026. The copyright holder for this preprint (which was not certified by peer review) is the author/funder, who has granted bioRxiv a license to display the preprint in perpetuity. It is made available under aCC-BY 4.0 International license.

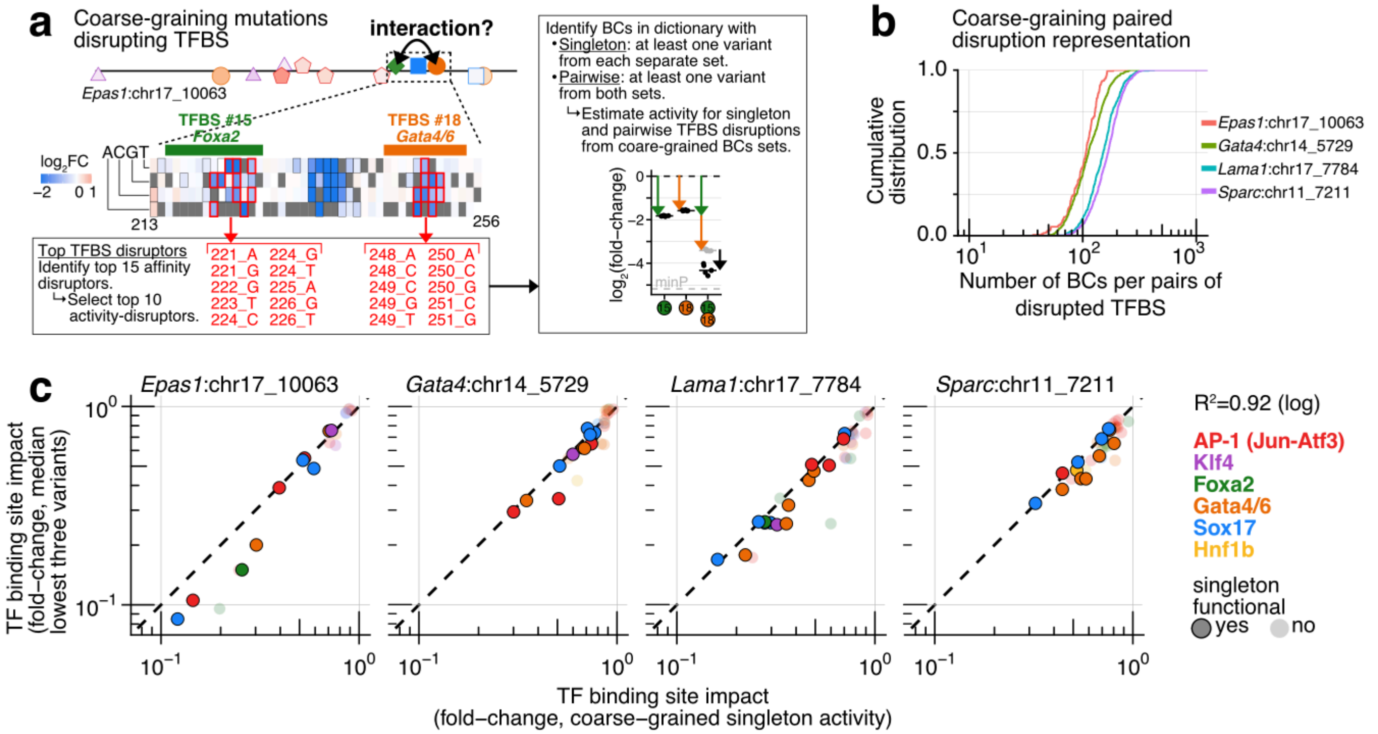

Supplementary Figure 14. Details of coarse-graining procedure for pairwise TFBS epistatic analysis. (a) Illustration of the procedure used to coarse-grain mutations at the level of full TFBSs to gather enough statistical power for epistatic mapping. In brief, for each TFBS, the top fifteen affinity-disrupting mutations were identified from the biophysical binding model, and the top ten activity-disrupting mutations among those were identified. All barcodes harbouring any of these TFBS disrupting variants were retained for activity estimates. For coarse-grained epistatic mapping, barcodes associated with at least one TFBS-disrupting variant from both binding sites in a pair were used for activity estimates. **(b)** Cumulative distribution of the number of BCs in the variant library associated with all pairs of disrupted TFBSs. **(c)** As a control for the coarse-graining procedure, the functional importances of single TFBSs were computed using the set of BCs identified above. Comparison of these coarse-grained ‘singleton’ binding site disruption estimates (x-axis) to the binding site impact as estimated by the median of the top three disrupting variants per position (from the full saturation mutagenesis, not coarse-grained) showed overall excellent agreement, albeit with the expected slight underestimation from coarse-graining.

bioRxiv preprint doi: https://doi.org/10.64898/2026.04.20.719625; this version posted April 21, 2026. The copyright holder for this preprint (which was not certified by peer review) is the author/funder, who has granted bioRxiv a license to display the preprint in perpetuity. It is made available under aCC-BY 4.0 International license.

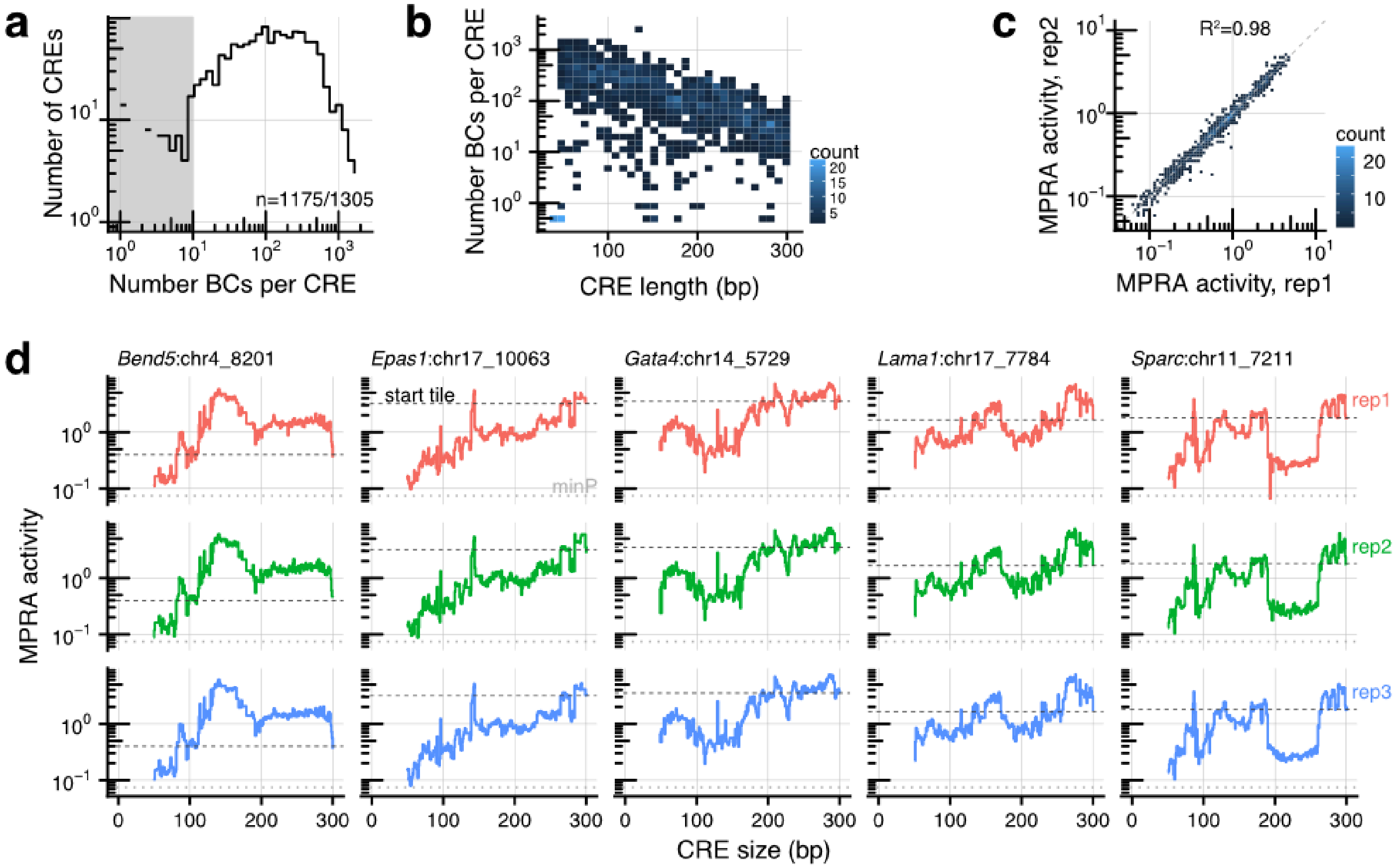

Supplementary Figure 15. Dictionary quality metrics and reproducibility of CRE-compactification MPRA data. (a) Distribution of number of BCs per compacted CRE (median 94 BCs per CRE; total = ~226,000; 1175 of 1305 CREs were represented by >10 BCs. **(b)** 2D heatmap highlighting a negative correlation between element size (CRE length in bp; x-axis) and representation in the library (# of BCs; y-axis), which led to a ~10-fold difference in the number of BCs for elements below 100 bp vs. above 250 bp. However, although shorter elements were more highly represented, very short tiles were strongly depleted due to gel-based size selection during cloning (*e.g.* all 35 tiles of length 46 bp or shorter had 0 BCs). **(c)** Example correlation in MPRA activities across biological replicates. We note that correlation is higher for this experiment presumably because of >20-fold more sequencing coverage compared to other experiments. **(d)** Direct visualization of the different compaction trajectories across replicates to highlight the biological as opposed to technical origin of the ruggedness in the data (y-axis: MPRA activity, x-axis: CRE size; a different model CRE is represented in each column).

bioRxiv preprint doi: https://doi.org/10.64898/2026.04.20.719625; this version posted April 21, 2026. The copyright holder for this preprint (which was not certified by peer review) is the author/funder, who has granted bioRxiv a license to display the preprint in perpetuity. It is made available under aCC-BY 4.0 International license.

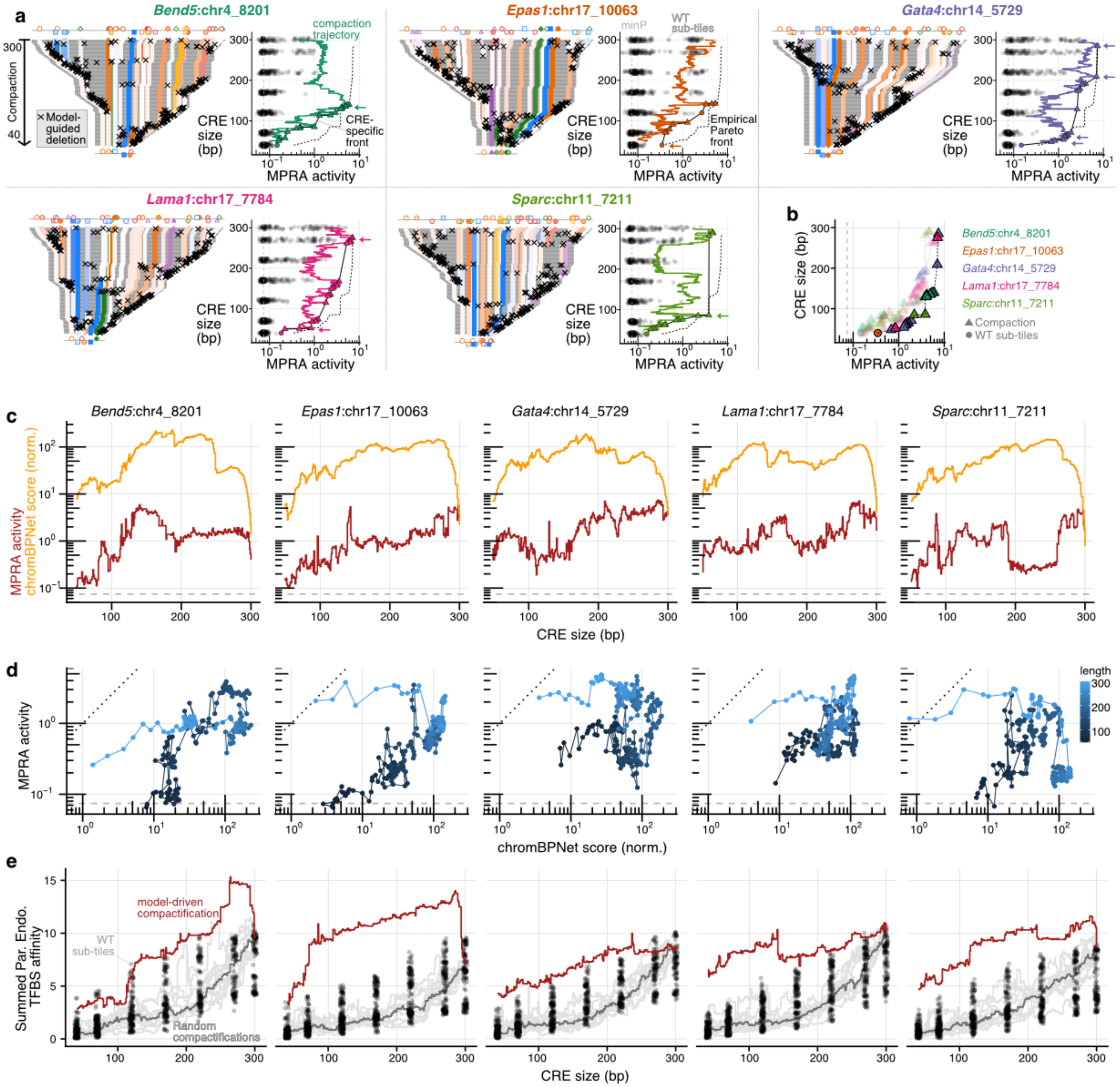

Supplementary Figure 16. Model-guided compaction of CREs reveals the trade-off between activity vs. length. (a) Similar to **Fig. 4a-b** but here for all 5 model CREs. **(b)** Reproduction of the global empirical Pareto front shown in **Fig. 4c** for reference. **(c)** Comparison of model accessibility scores (chromBPNet, orange) vs. activities (MPRA, dark red) along compaction trajectories. Model scores are normalized so that the average score of background sequences is the same as experimentally measured minP value. **(d)** Replotting of the data shown in panel **c**, but here comparing the model accessibility scores (x-axis: chromBPNet) vs. activities (y-axis: MPRA) directly, and encoding CRE length with color (light to dark blue: long to short). Dashed line represents the 1:1 line. Substantial correlation (R^2) is observed for the 3 of 5 elements:: *Bend5*: 0.40; *Epas1*: 0.37; *Gata4*: 0.0026; *Lama1*: 0.16; *Sparc*: 0.27. **(e)** As a measure for the number of TFBS within the sequences, y-axes shows quantification of summed normalized affinity to parietal endoderm CRM TFBSs for model-guided compaction trajectory sequences (dark red), wild-type endogenous tile sequences (points with x-jitter), and sequences from ten random deletion trajectories (*in silico*, not tested experimentally; light gray are individual trajectories, dark gray is median across the 10 trajectories). Model-guided compaction trajectory sequences show elevated TFBS densities compared to both WT endogenous subtile sequences and random deletion trajectory sequences.

bioRxiv preprint doi: https://doi.org/10.64898/2026.04.20.719625; this version posted April 21, 2026. The copyright holder for this preprint (which was not certified by peer review) is the author/funder, who has granted bioRxiv a license to display the preprint in perpetuity. It is made available under aCC-BY 4.0 International license.

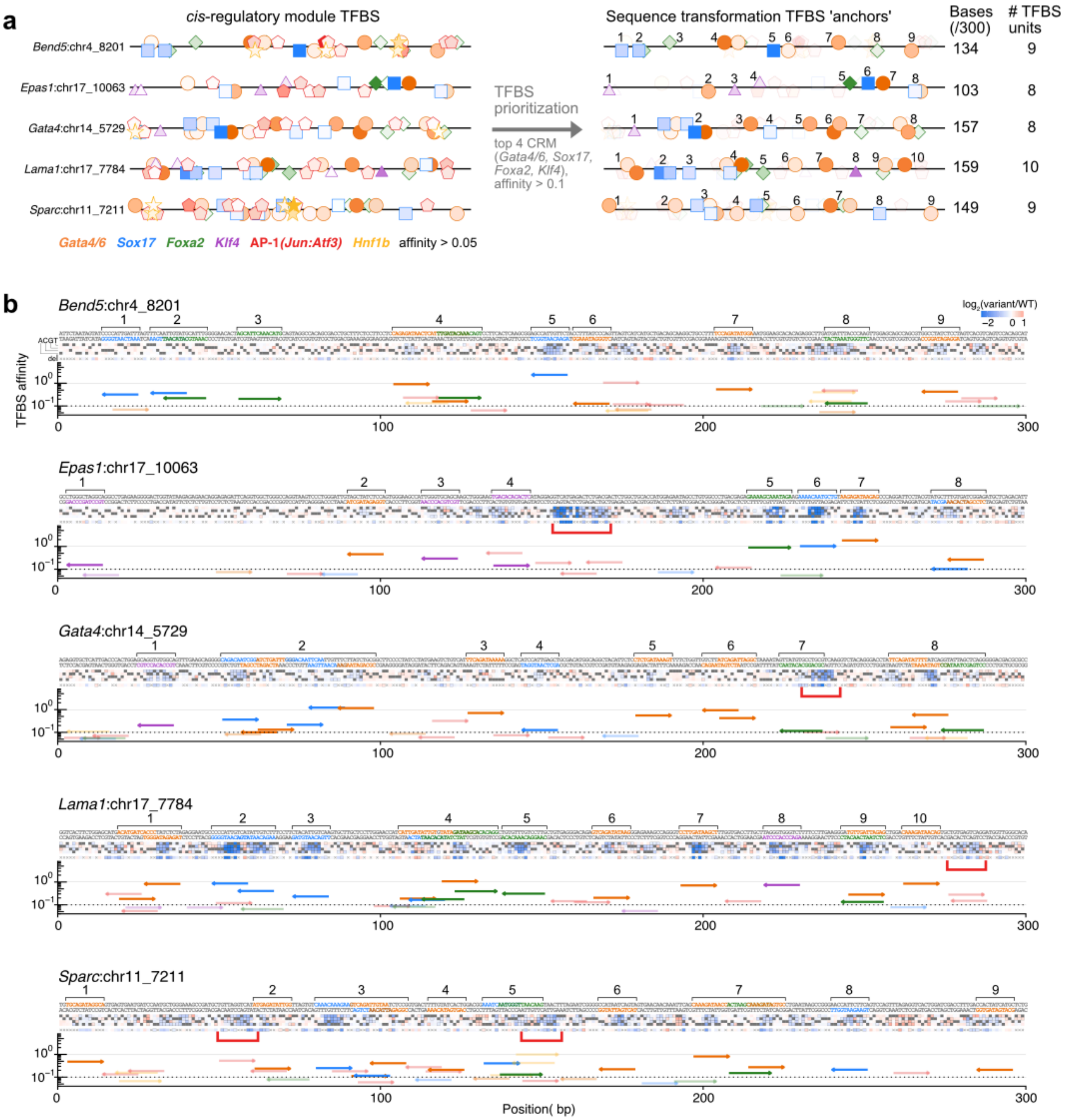

Supplementary Figure 17. Details on the selection of anchor TFBS units for enhancer derivatization. (a) Illustration of the selection process for the definition of TFBS anchors for the derivatization process. To limit the total covered sequence by anchors out of all putative identified CRM TFBS (left, norm. affinity>0.05), the following criteria was applied (right, non-selected TFBS shaded): (1) the top four TFs associated with the highest differential accessibility (Gata4/6, Sox17, Foxa2, and Klf4, **Fig. S1b**) were selected and (2) a threshold of normalized affinity of 0.1 was chosen. These defined TFBSs which were then joined if they overlapped into indivisible units for the derivatization process. Of note, we did not rely on singleton-functional definitions or our saturation mutagenesis data to make our selection. Derivatization anchors cover between 103 and 159 out of 300 bp, and include between 8 and 10 units (some composed of multiple overlapping TFBSs). **(b)** Illustration of the identified anchors (numbered as in panel a) contextualized with the saturation mutagenesis data (heatmap, same as **Fig. 3**, **S9**) and TFBS affinities (arrows indicate directionality of the sites). Red brackets identify AP-1 TFBSs with strong functional impact that were not selected for our procedure (see **Fig. S24** on analysis of synthetic thripsis for additional context).

bioRxiv preprint doi: https://doi.org/10.64898/2026.04.20.719625; this version posted April 21, 2026. The copyright holder for this preprint (which was not certified by peer review) is the author/funder, who has granted bioRxiv a license to display the preprint in perpetuity. It is made available under aCC-BY 4.0 International license.

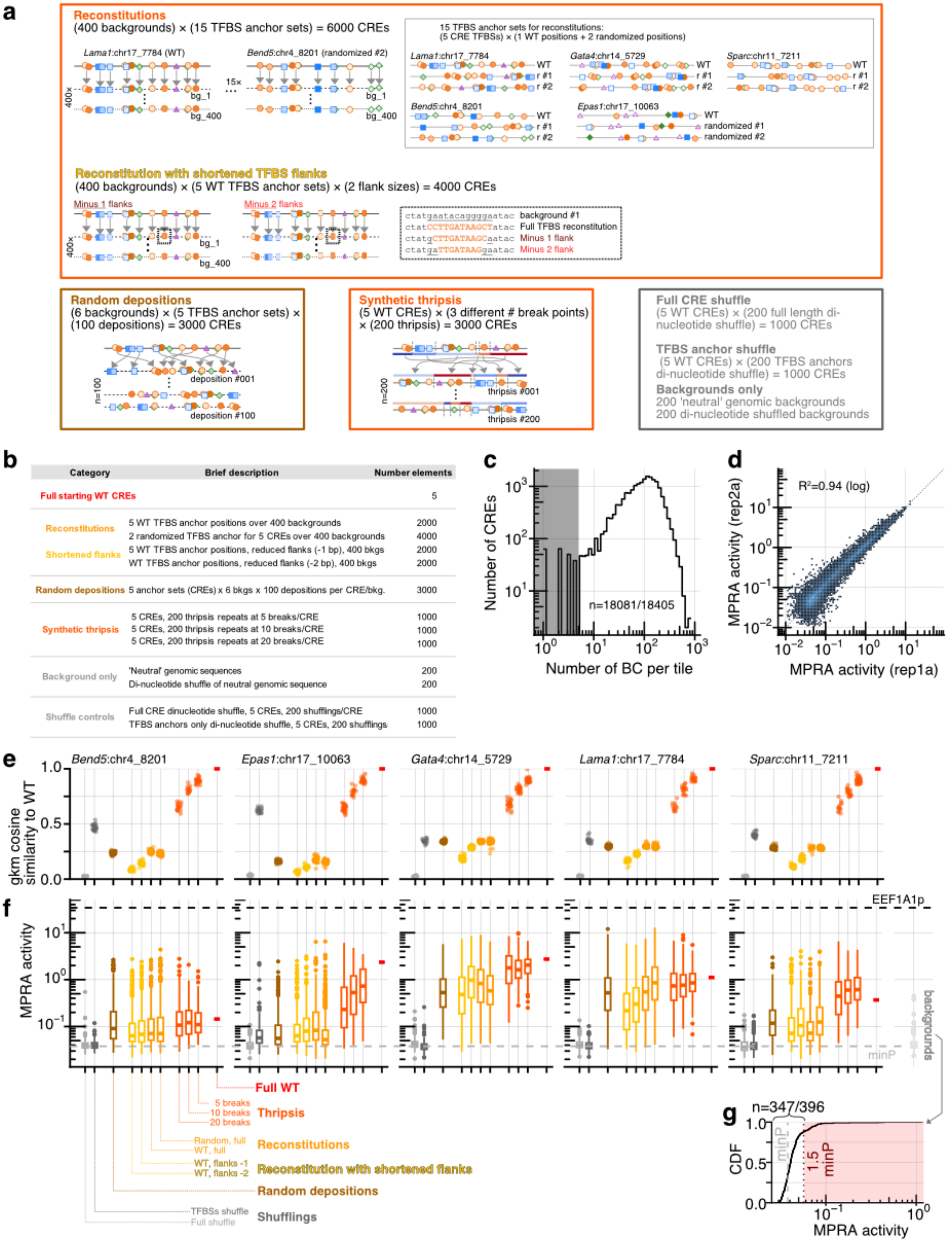

Supplementary Figure 18. Composition of tested enhancer derivatives and quality metrics of library/MPRA
(legend on next page)

bioRxiv preprint doi: https://doi.org/10.64898/2026.04.20.719625; this version posted April 21, 2026. The copyright holder for this preprint (which was not certified by peer review) is the author/funder, who has granted bioRxiv a license to display the preprint in perpetuity. It is made available under aCC-BY 4.0 International license.

Supplementary Figure 18. Composition of tested enhancer derivatives and quality metrics of library/MPRA. (a) Schematics of the different classes of derivatization and controls included in the derivatization library. Each category of sequence is boxed. Reconstitutions consisted in 400 background DNAs × 5 sets of anchor sites (one per model CRE) × 3 anchor positions (original n=2000; two randomized and fixed n=4000). Schematic of reconstituted TFBS positions (yellow) are shown at right. To test for the importance of flanking bases, the WT TFBS positions reconstitutions were also implemented with -1 and -2 bases from the flanks of the anchor sites (taken to be the full extent of the quantitative model used, ranging in size from 12 to 14 bp), n=4000. Inset shows an example Gata4/6 binding site with -1 (10 bp TFBS deposited) and -2 bp on both sides (8 bp TFBS deposited). Random depositions (n=3000, brown) were performed on 6 fixed background DNAs × 5 set of anchor sites (one per model CRE) × 100 random anchor depositions. Importantly, the same deposition positions were fixed across backgrounds for a given CRE. Thrispis (n=3000) was performed 200 times on 5 sets of anchor sites (one per CRE) with three number of break points (5, 10, and 20 breaks). The following negative controls were also included: (1) backgrounds alone used for reconstitution and random deposition were also tested (n=200 genomic neutral, n=200 ni-nucleotide shuffled), (2) n=200 full dinucleotide shuffle per starting CREs (n=1000), and (3) n=200 dinucleotide shuffle of just the anchor sites per CRE (n=1000). The starting WT sequences were also profiled, leading to a total of 18405 CREs of 300 bp. **(b)** Summary table of the number of CRE per class. **(c)** Representation of the CRE derivatives in the MPRA library: distribution of number of BCs per element (98.2%, or 18081/18405, of the synthesized sequences were represented with ≥5 BCs, 1.89M BCs total, median coverage 91 BC/CRE). **(d)** Representative correlation between replicates for the MPRA data (R^2 on log-transformed data = 0.94). **(e)** Comparison of gapped k-mer composition[91] (size l=11 with k=7 non-gapped position) between enhancer derivatives and their respective wild-type quantified as the cosine similarity between WT and derivative enhancer gkm vectors. Similarities for a sampling of 400 derivatives per starting CREs were computed. **(f)** Box plot summarizing the activity of all classes of derivatization and controls (color aligned with panel **a** categories), faceted by model CRE (left to right panels). Spiked-in minP (basal) only and EEF1A1 (positive) controls are shown as grey and black dashed lines respectively. Background only sequences (light grey) are shown to the right. Full di-nucleotide shuffling of CREs displayed overall low activity (n=887/1000 from 87% to 93% of full di-shufflings per CRE with <1.5-fold minP). Furthermore, di-nucleotide shufflings of only the anchor sites also broadly abrogated activity in all (<1.5-fold minP, from 88% to 96% of anchor shuffling per CRE) but CRE *Epas1*:chr17_10063 (<1.5-fold minP for 50% of anchor shuffling), presumably because of the functionally important cluster of AP-1 binding sites left unperturbed in this process (**Fig. S17**). **(g)** Cumulative distribution of MPRA activity of the background sequences, which had had overwhelmingly low activity over our basal control. With a threshold of 1.5 above minP, n=347/396 (4 background sequences were not represented in our final library) of background sequences displayed no substantial autonomous activity.

bioRxiv preprint doi: https://doi.org/10.64898/2026.04.20.719625; this version posted April 21, 2026. The copyright holder for this preprint (which was not certified by peer review) is the author/funder, who has granted bioRxiv a license to display the preprint in perpetuity. It is made available under aCC-BY 4.0 International license.

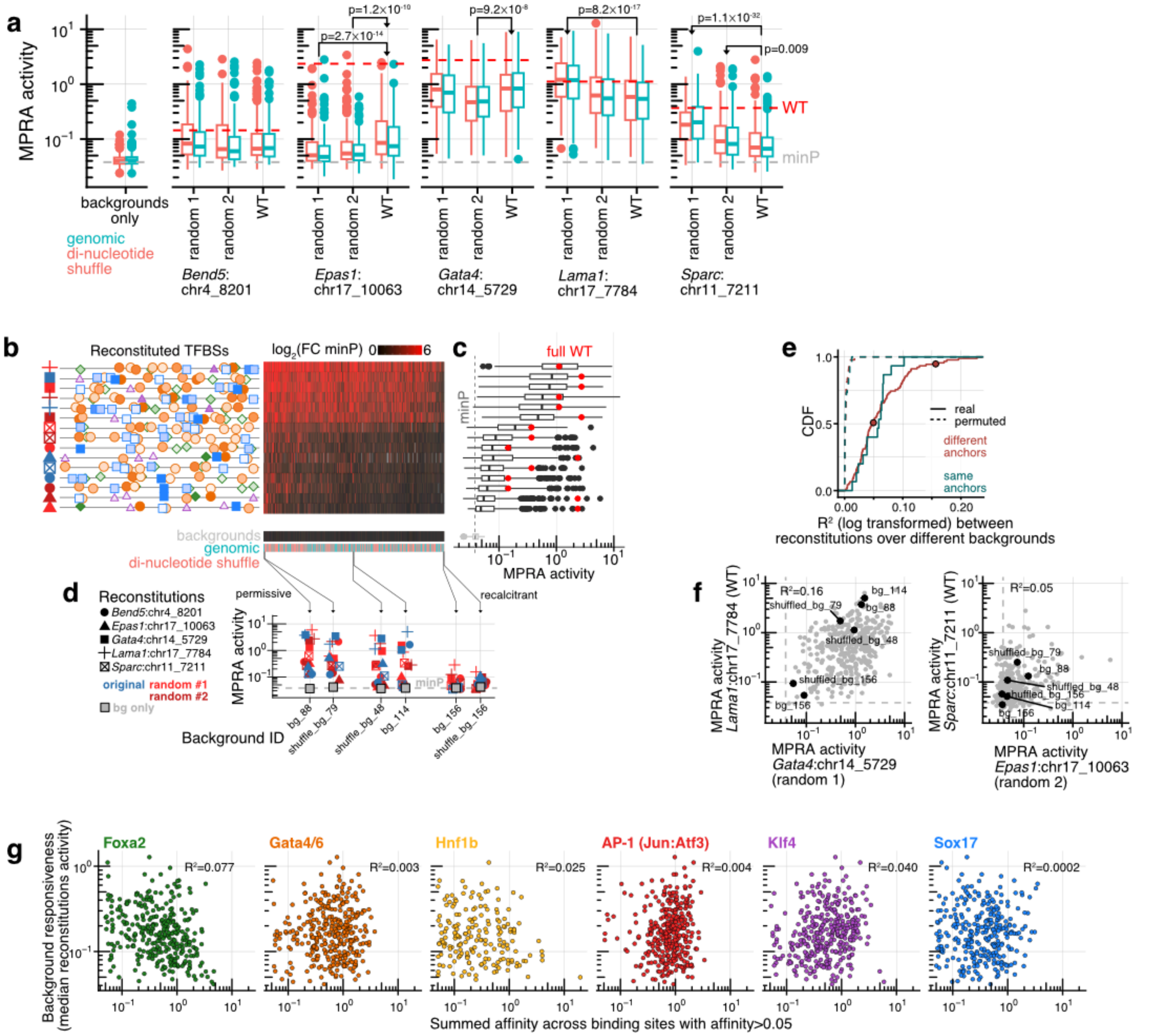

Supplementary Figure 19. Highly variable background 'responsiveness' to TFBS reconstitutions. (a) Box plot showing the MPRA activity of different reconstitutions and negative controls. Background-only activities (leftmost) are stratified by sequences sampled from putatively neutral regions of the mouse genome ('genomic', cyan, n=200) and di-nucleotide shufflings of these sequences ('di-nucleotide shuffle', light red, n=200). There is no difference in overall activity between the two categories (p=0.2 two-sided rank-sum test). Each panel corresponds to reconstitutions from different model CREs, and each model CRE has three depositions (WT positions, and two randomized positions). There was no significant difference in overall responsiveness from genomic or shuffled versions of the backgrounds. Instances with significant differences (one-sided rank-sum test with Bonferroni correction p-value shown) are indicated by the black arrow (e.g., *Lama1*:chr17_7784 randomized 1 more potently inducing activity than its WT counterpart). minP basal activity is shown as dashed grey line. **(b)** Headmap of $\log_2$ fold-change over minP basal activity (black: low activity, red: high activity) summarizing the full dataset on reconstitutions for the 347 non-autonomously active background sequences (see **Fig. S18g**). Rows are organized according to depositions, with the schematic of the anchor TFBS shown on the left, with

bioRxiv preprint doi: https://doi.org/10.64898/2026.04.20.719625; this version posted April 21, 2026. The copyright holder for this preprint (which was not certified by peer review) is the author/funder, who has granted bioRxiv a license to display the preprint in perpetuity. It is made available under aCC-BY 4.0 International license.

the origin following the legend of panel **d** (see also **Fig. S18a**). Columns correspond to different background sequences, which are ordered from most (left) to least (right) responsive. Data for background sequences alone are shown at bottom in a separate line. **(c)** Boxplot for the activity for all backgrounds corresponding to the reconstitution of the aligned rows. Full WT activity for the underlying model CRE is shown as a red dot. **(d)** Examples of permissive, intermediate, and recalcitrant background sequences (arrows indicating identities in heatmap of panel **b**). Even though all are equally autonomous inactive, their response to adding TFBS varies drastically. **(e)** Cumulative distribution of correlations (R^2 on log-transformed activities) for all possible pairs (15 choose 2 = 105) of reconstitutions across the backgrounds. Pairs from anchors from different model CREs are shown in red, and those from the same model CRE in cyan. Dashed lines show permuted samples comparisons. Points mark the two examples shown in panel **f**. (f) Example pairs showing underlying data to the correlations in panel **e**. Backgrounds shown in panel **d** are marked by black dots. **(g)** Correlations between background sequences pre-existing parietal endoderm CRM TFBS (calculated as the summed affinity of all mapped TFBS with normalized affinity > 0.05) and median responsiveness (across all 15 reconstitutions).

bioRxiv preprint doi: https://doi.org/10.64898/2026.04.20.719625; this version posted April 21, 2026. The copyright holder for this preprint (which was not certified by peer review) is the author/funder, who has granted bioRxiv a license to display the preprint in perpetuity. It is made available under aCC-BY 4.0 International license.

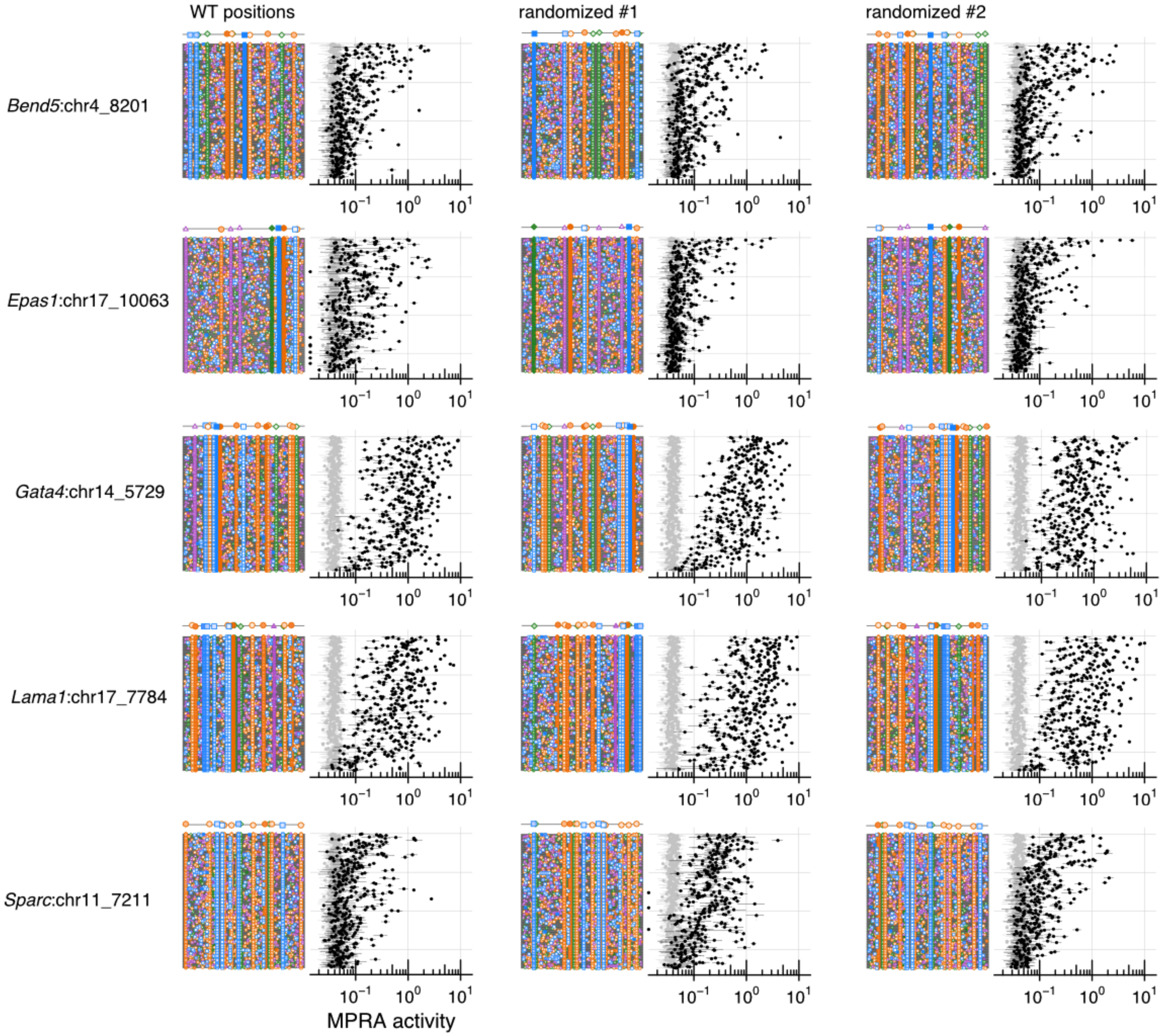

Supplementary Figure 20. TFBS anchors reconstituted across hundreds of 'neutral' DNA background sequences. Reconstitution dataset, equivalent of **Fig. 5b-c** (only showing the reconstitution panels), but for all 15 configurations of reconstitutions (5 model CRE TFBS anchors across 3 sets of positions: one WT and two randomized), organized by model CREs (rows) and reconstituted positions (columns). All background sequences are ordered from top to bottom in the same way across panels, as determined by the overall responsiveness across reconstitutions (top: high response, bottom: low response). Only non-autonomously active backgrounds (activity <1.5 minP basal activity) are included for these plots (n=347/400).

bioRxiv preprint doi: https://doi.org/10.64898/2026.04.20.719625; this version posted April 21, 2026. The copyright holder for this preprint (which was not certified by peer review) is the author/funder, who has granted bioRxiv a license to display the preprint in perpetuity. It is made available under aCC-BY 4.0 International license.

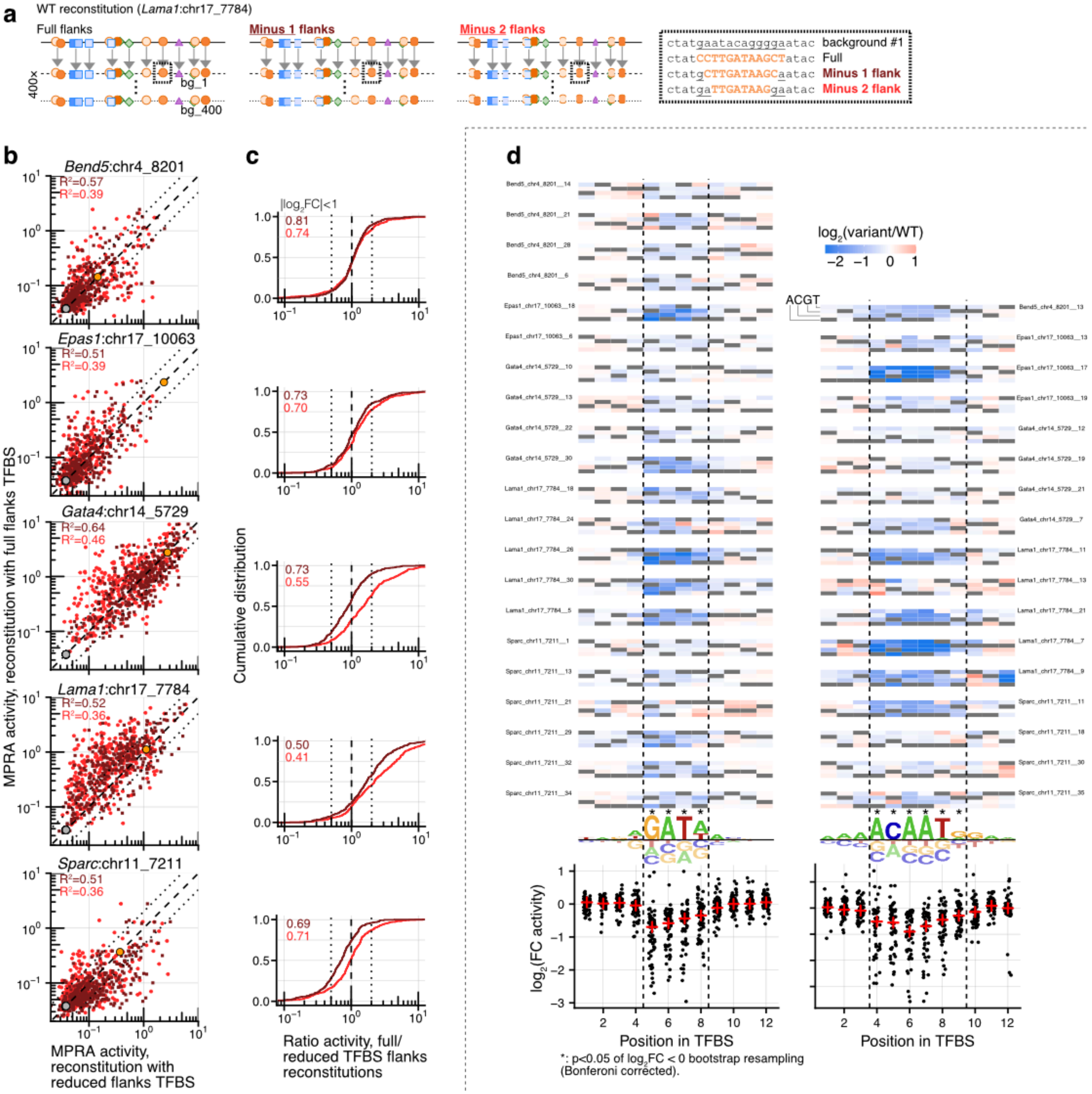

Supplementary Figure 21. Modest importance of 12-14 bp TFBS flanks for reconstitution activity. Given the importance of bases flanking the core motifs in TF binding[5,89], a natural explanation for difference in reconstitution activities would be that the different backgrounds lead to small-magnitude modulation in affinity of the anchor sites as a result of changing surrounding sequences. To explicitly test this, reconstitutions with reduced flanks were performed (with WT TFBS anchor positions). **(a)** Schematic of reconstitution with TFBS of reduced flanks. We tested reconstitutions with reduced flanks by removing one (minus 1 flanks) and two (minus 2 flanks) base pairs from both ends of the TFBS (5 model CREs across 400 backgrounds, n = 4000 sequences). The rightmost dashed-line box shows an example Gata4/6 binding site with the full vs reduced flanks sequences deposited in the background. **(b)** Correlation between activity of full reconstitutions (y axis) vs. reduced flanks (x axis, dark red: minus 1, light red: minus 2). R^2 of log-transformed values are shown in the plot. Dashed and dotted lines show the 1:1 and two-fold deviations respectively. **(c)** Cumulative distribution

bioRxiv preprint doi: https://doi.org/10.64898/2026.04.20.719625; this version posted April 21, 2026. The copyright holder for this preprint (which was not certified by peer review) is the author/funder, who has granted bioRxiv a license to display the preprint in perpetuity. It is made available under aCC-BY 4.0 International license.

of ratio of activities between full and reduced. Proportion of reconstitutions with less than 2-fold difference between full and reduced indicated on the plot. **(d)** Correlations are substantial between full and reduced, they remain incomplete, suggesting flanks could indeed substantially contribute. As another line of evidence against this possibility, we aligned our saturation mutagenesis data for the most prevalent singleton-functional TFs (Gata4/6: left, and Sox17: right). Top of panel shows saturation mutagenesis heatmap (log_2FC vs. WT, as in **Fig. 3**), one sub-panel per TFBS, with CRE and TFBS ID indicated. The bottom of panels shows aggregated log_2FC (bottom) as a function of position within the TFBS (horizontal jitter for visualization, median shown as red +). Significant effects ($log_2FC<0$ in resampling bootstrap at $p<0.05$ with Bonferroni correction) are indicated by *. Probound[74] binding model weights are shown as a sequence logo. The empirically determined core region is delimited by vertical dashed lines. Within our saturation mutagenesis data, flanking positions not included in the reduced reconstitutions indeed do not contribute substantially to activity. Consistently with the correlations in background responsiveness across reconstitutions, these point to complex sequence determinants beyond the flanks of TFBS anchors.

bioRxiv preprint doi: https://doi.org/10.64898/2026.04.20.719625; this version posted April 21, 2026. The copyright holder for this preprint (which was not certified by peer review) is the author/funder, who has granted bioRxiv a license to display the preprint in perpetuity. It is made available under aCC-BY 4.0 International license.

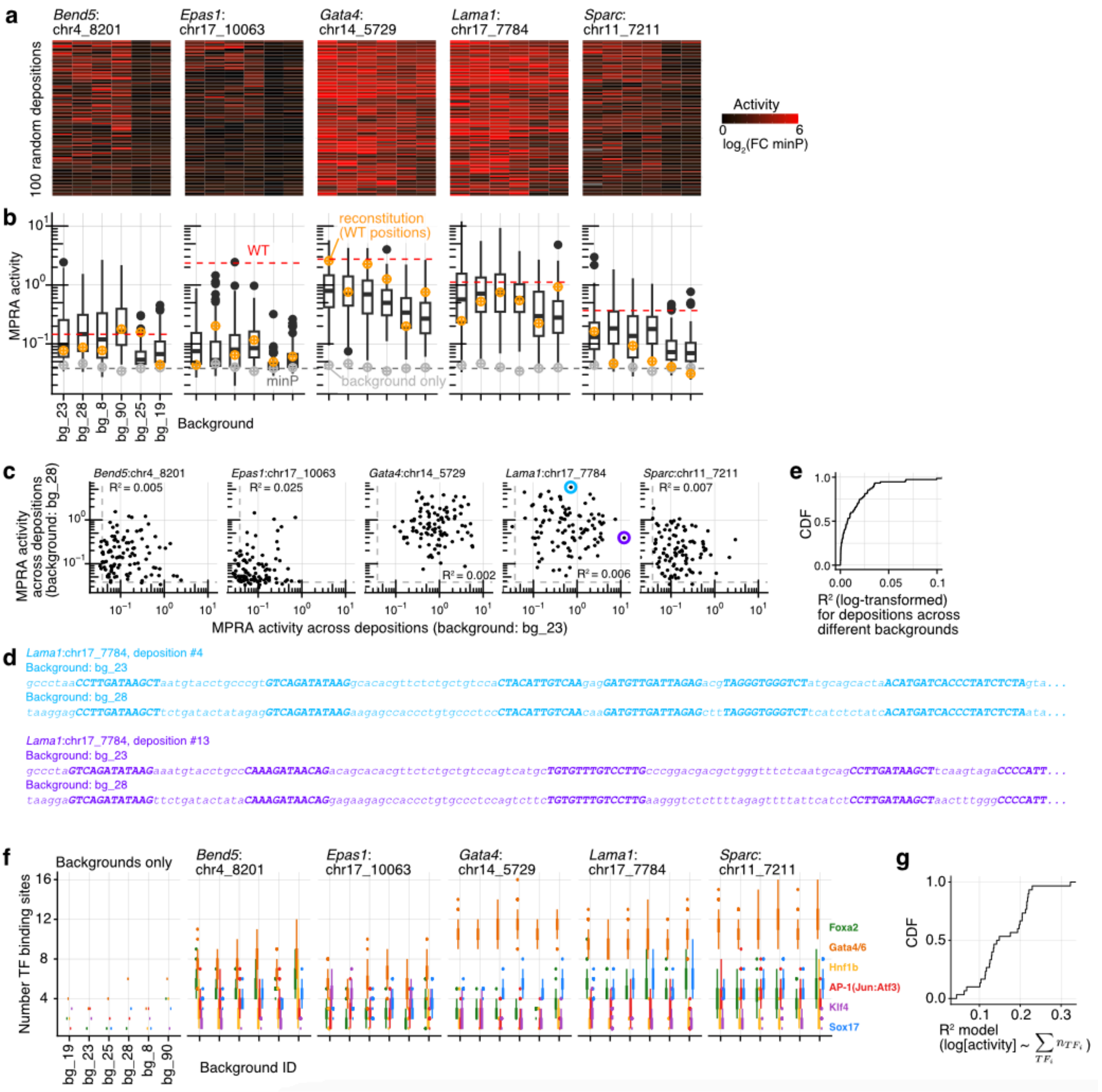

Supplementary Figure 22. Additional analysis of random TFBS depositions. (a) Heatmaps (one per model CRE) of random deposition activities (log_2FC over minP) over deposition positions (rows, fixed across backgrounds internally for each CRE) and background DNA (columns). Deposition and backgrounds are ordered by activities. **(b)** Boxplot aggregating the data over deposition from panel **a**. minP baseline is shown as gray dashed line and WT CRE activity as a red dashed line. Background-only activity is shown as gray + and WT TFBS anchors reconstituted (equivalent to a random deposition with the WT positions) in the same background as orange +. Note how the WT reconstitutions fall across the full range of activities of random depositions (see **Fig. 5g**). **(c)** Example of correlation (R^2 on log-transformed) between two background DNA (bg_23 vs. bg_28) across 100 depositions for 5 CREs. Correlations are overall low. Colored dots in *Lama1*:chr17_7784 CRE indicate examples with strongly discordant activities across backgrounds shown in panel **d**. **(d)** First 145 bp of sequences for CREs highlighted in panel **c** (bg_23 and bg_28 with depositions #4 and #13)

bioRxiv preprint doi: https://doi.org/10.64898/2026.04.20.719625; this version posted April 21, 2026. The copyright holder for this preprint (which was not certified by peer review) is the author/funder, who has granted bioRxiv a license to display the preprint in perpetuity. It is made available under aCC-BY 4.0 International license.

with concordant coloring. Random depositions are fixed, but background sequences differ. TFBS anchors are shown bolded and in capital letters (background, lowercase). **(e)** Cumulative distribution of R^2 across pairs of background/CRE pairs (6 choose 2 = 15 pairs with 5 CREs). **(f)** Distribution of mapped number of TFBS (>0.1 normalized affinity) across random depositions stratified by CREs and backgrounds. Boxplot illustrates the slight variability in the number of binding sites across depositions putatively due to difference in chimeric sequences at junctions of TFBS and background. On average, we observe changes between 1.5 to 2.9 binding sites (span of the central 80% range) per CRE per TF over a fixed anchor set and background. **(g)** Simple linear models (one per CRE/background pair) relating these slight TFBS number variations to activity hold limited predictive potential (average R^2 of <0.2), underscoring that 'chimeric' TFBS are probably a minor determinant of activity.

bioRxiv preprint doi: https://doi.org/10.64898/2026.04.20.719625; this version posted April 21, 2026. The copyright holder for this preprint (which was not certified by peer review) is the author/funder, who has granted bioRxiv a license to display the preprint in perpetuity. It is made available under aCC-BY 4.0 International license.

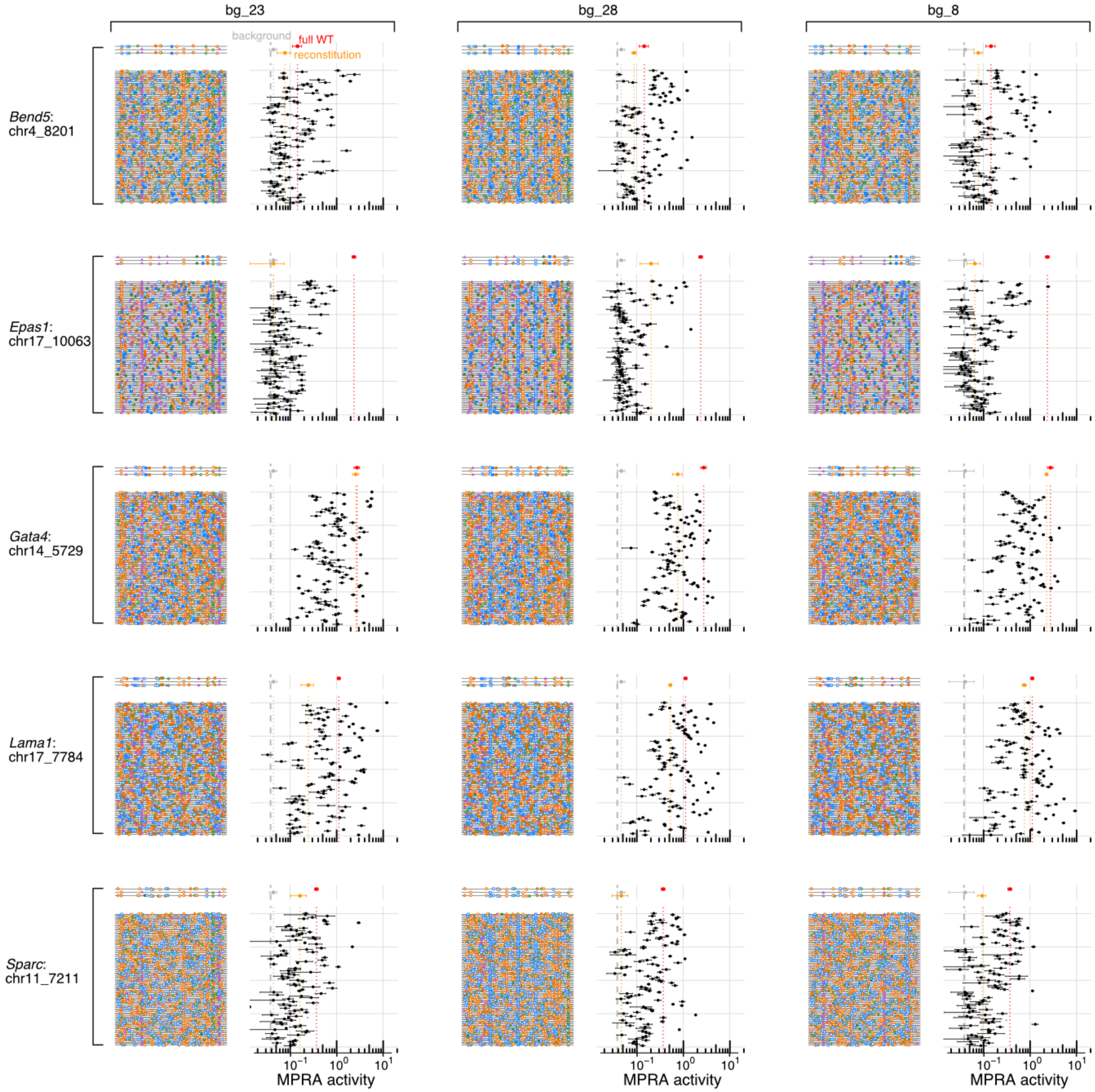

Supplementary Figure 23. Thousands of random TFBS depositions across 5 CREs × 6 backgrounds (part 1)
Random deposition dataset, analogous to **Fig. 5e-f**, but on the full set of 30 CRE/background pairs. Depositions (fixed across backgrounds for each model CREs) are ordered based on overall activity.

bioRxiv preprint doi: https://doi.org/10.64898/2026.04.20.719625; this version posted April 21, 2026. The copyright holder for this preprint (which was not certified by peer review) is the author/funder, who has granted bioRxiv a license to display the preprint in perpetuity. It is made available under aCC-BY 4.0 International license.

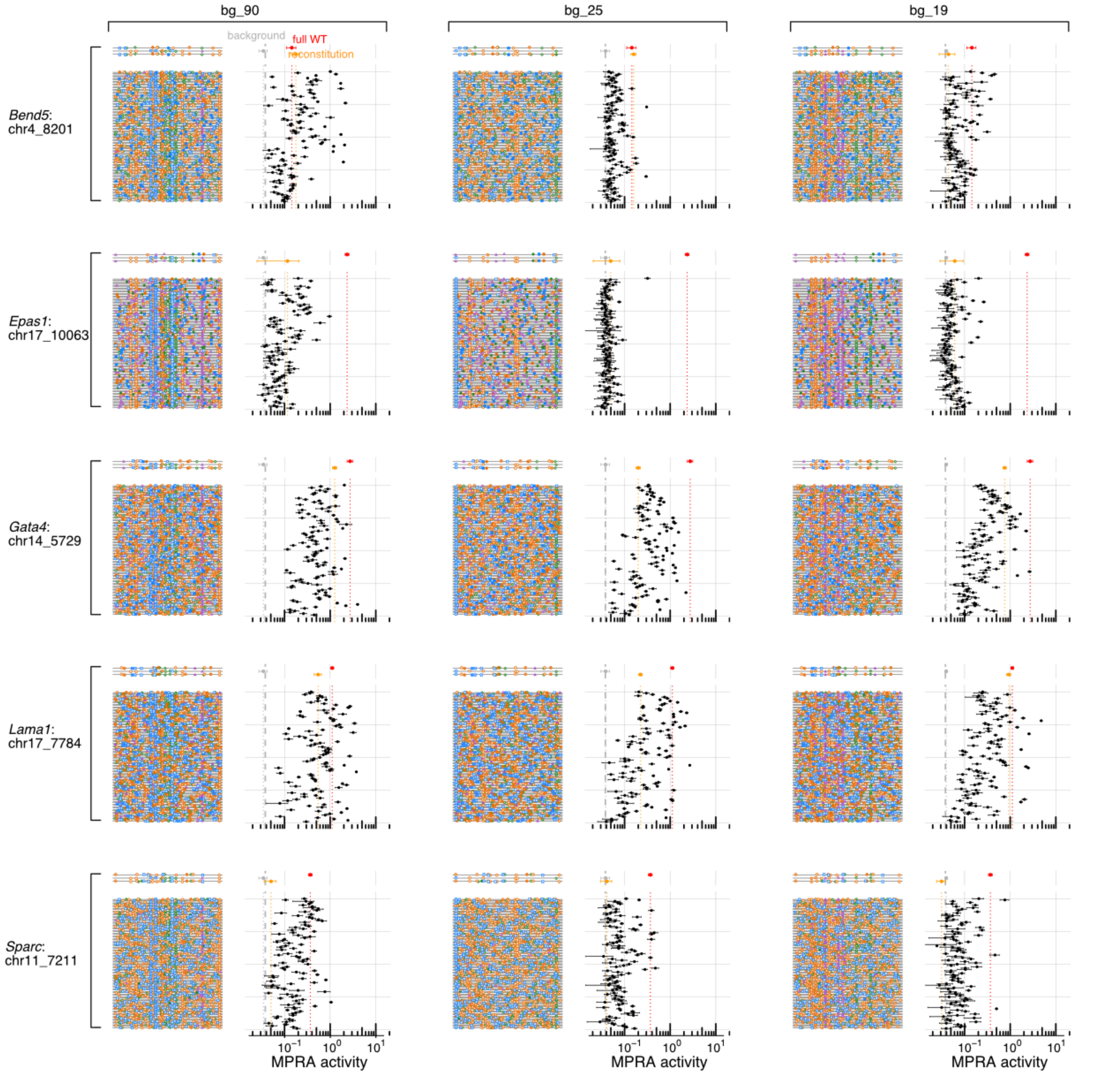

Supplementary Figure 23. Thousands of random TFBS depositions across 5 CREs × 6 backgrounds (part 2). Random deposition dataset, analogous to **Fig. 5e-f**, but on the full set of 30 CRE/background pairs. Depositions (fixed across backgrounds for each model CREs) are ordered based on overall activity.

bioRxiv preprint doi: https://doi.org/10.64898/2026.04.20.719625; this version posted April 21, 2026. The copyright holder for this preprint (which was not certified by peer review) is the author/funder, who has granted bioRxiv a license to display the preprint in perpetuity. It is made available under aCC-BY 4.0 International license.

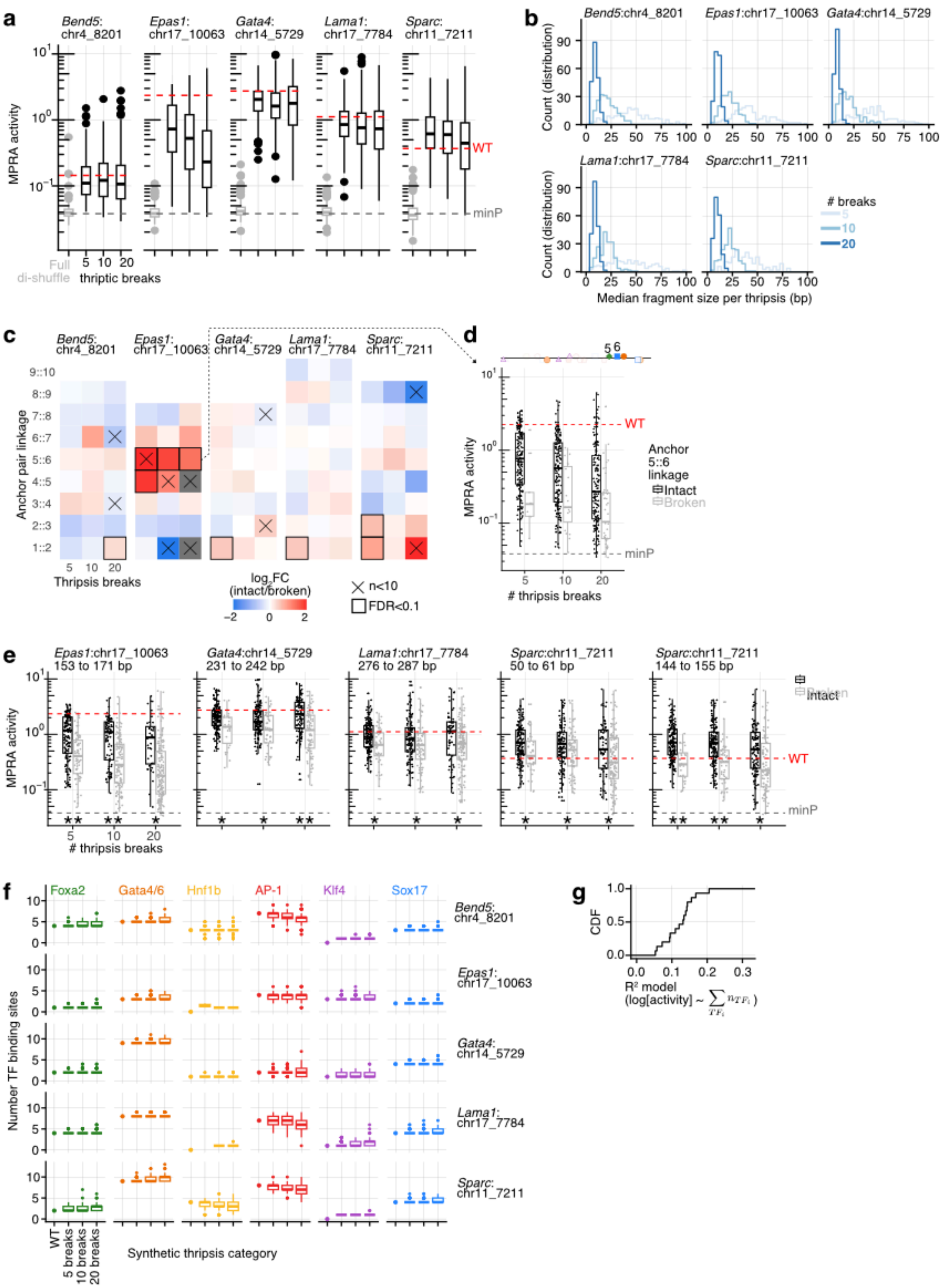

Supplementary Figure 24. Analysis of synthetic thripsis derivatives. (a) Box plot of CRE derivatives MPRA activity stratified by model CRE (panels) and number of thriptic break points (x-axis). For reference, the full CRE di-nucleotide shufflings for each CREs are included (gray box plot), minP-only (gray dashed line) and corresponding model CRE WT (red dashed line). **(b)** Distribution of median fragment length resulting from the synthetic thripsis operation per CRE. The

bioRxiv preprint doi: https://doi.org/10.64898/2026.04.20.719625; this version posted April 21, 2026. The copyright holder for this preprint (which was not certified by peer review) is the author/funder, who has granted bioRxiv a license to display the preprint in perpetuity. It is made available under aCC-BY 4.0 International license.

average median fragment size per thripsis ranged from 45 to 8 bp from 5 to 20 break points. This is slightly shorter than the expected *L/(n+1)* because of the anchor-avoiding constraint. **(c)** For each consecutive pairs of TFBS anchors, the thripsis CREs were split between those preserving or not the linkage (i.e., with or without one or more break point between them) and the difference in activity quantified. FC between medians of intact/broken linkage is shown as a heatmap. Linkages with FDR<0.1 (rank-sum p-value with Benjamini-Hochberg correction) are highlighted with dark edges. Linkages with fewer than 10 observations are marked by an ✕ (and those with none, e.g., for anchors that are far apart, are grayed out). Only the TFBS 5::6 linkage in CRE *Epas1*:chr17_10063 is significant across the three thripsis conditions (FDR<0.03) **(d)** Underlying data for the 5::6 linkage data pointed at (dashed line with arrow) in panel **c**. Each point is the activity of a thripsis CRE, split between linkage preserving [black] and linkage abrogating [gray] instances. **(e)** Similar analysis as panels c-d, but now with functionally important AP-1 TFBS (not included in the anchor set, indicated in **Fig. S17b**). Thripsis activities are stratified by whether the indicated region is intact (black) or not (gray). Integrity of the regions are all significantly related to activity (ranksum test with Benjamini-Hochberg correction, *: 0.0005 ≤ FDR < 0.05, **: FDR < 0.0005). **(f)** Quantification of the number of CRM TFBS (columns) per CRE (normalized affinity > 0.1) across different model CREs (rows) organized by different number of thriptic break points shown as a boxplot. These provide information about the likelihood of chimeric junctions creating new TFBS. Quantification for WT model CREs are included as a single point. Overall, median change in the number of TFBS is low (5 breaks: 0.5, 10 breaks: 0.8, 20 breaks: 1.3). (g) These changes in TFBS numbers can be used to predict log-activity with a linear model, but they hold corresponding little explanatory power (on average R^2<0.13). Shown is the distribution of R^2 for the 15 situations (5 model CREs by 3 thripsis set).

bioRxiv preprint doi: https://doi.org/10.64898/2026.04.20.719625; this version posted April 21, 2026. The copyright holder for this preprint (which was not certified by peer review) is the author/funder, who has granted bioRxiv a license to display the preprint in perpetuity. It is made available under aCC-BY 4.0 International license.

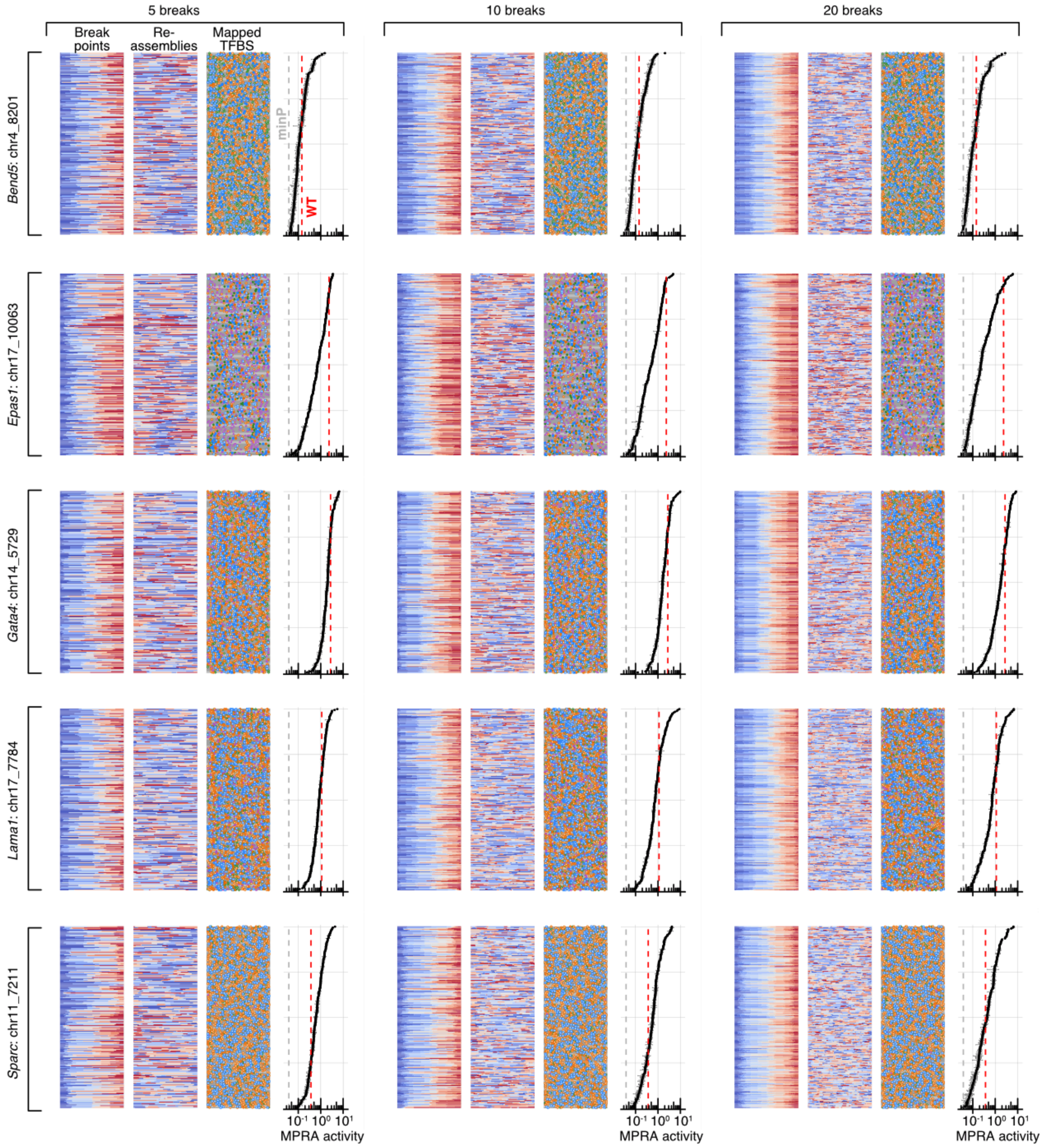

Supplementary Figure 25. Thousands of synthetic thripsis from five model CREs. Analogous to **Fig. 5i-j**, but for all different numbers of thriptic fragments and starting CREs. Since comparisons were not possible across thripsis (unlike for reconstitutions/random depositions), thripsis operations are ordered vertically by activity.
